# Search for low-mass dilepton resonances in Higgs boson decays to four-lepton final states in proton–proton collisions at $\sqrt{s}=13\,\text {TeV}$

**DOI:** 10.1140/epjc/s10052-022-10127-0

**Published:** 2022-04-04

**Authors:** A. Tumasyan, W. Adam, T. Bergauer, M. Dragicevic, J. Erö, A. Escalante Del Valle, R. Frühwirth, M. Jeitler, N. Krammer, L. Lechner, D. Liko, T. Madlener, I. Mikulec, F. M. Pitters, N. Rad, J. Schieck, R. Schöfbeck, M. Spanring, S. Templ, W. Waltenberger, C.-E. Wulz, M. Zarucki, V. Chekhovsky, A. Litomin, V. Makarenko, J. Suarez Gonzalez, M. R. Darwish, E. A. De Wolf, D. Di Croce, X. Janssen, T. Kello, A. Lelek, M. Pieters, H. Rejeb Sfar, H. Van Haevermaet, P. Van Mechelen, S. Van Putte, N. Van Remortel, F. Blekman, E. S. Bols, S. S. Chhibra, J. D’Hondt, J. De Clercq, D. Lontkovskyi, S. Lowette, I. Marchesini, S. Moortgat, A. Morton, Q. Python, S. Tavernier, W. Van Doninck, P. Van Mulders, D. Beghin, B. Bilin, B. Clerbaux, G. De Lentdecker, B. Dorney, L. Favart, A. Grebenyuk, A. K. Kalsi, I. Makarenko, L. Moureaux, L. Pétré, A. Popov, N. Postiau, E. Starling, L. Thomas, C. Vander Velde, P. Vanlaer, D. Vannerom, L. Wezenbeek, T. Cornelis, D. Dobur, M. Gruchala, I. Khvastunov, M. Niedziela, C. Roskas, K. Skovpen, M. Tytgat, W. Verbeke, B. Vermassen, M. Vit, G. Bruno, F. Bury, C. Caputo, P. David, C. Delaere, M. Delcourt, I. S. Donertas, A. Giammanco, V. Lemaitre, K. Mondal, J. Prisciandaro, A. Taliercio, M. Teklishyn, P. Vischia, S. Wertz, S. Wuyckens, G. A. Alves, C. Hensel, A. Moraes, W. L. Aldá Júnior, E. Belchior Batista Das Chagas, H. BRANDAO MALBOUISSON, W. Carvalho, J. Chinellato, E. Coelho, E. M. Da Costa, G. G. Da Silveira, D. De Jesus Damiao, S. Fonseca De Souza, J. Martins, D. Matos Figueiredo, M. Medina Jaime, C. Mora Herrera, L. Mundim, H. Nogima, P. Rebello Teles, L. J. Sanchez Rosas, A. Santoro, S. M. Silva Do Amaral, A. Sznajder, M. Thiel, F. Torres Da Silva De Araujo, A. Vilela Pereira, C. A. Bernardes, L. Calligaris, T. R. Fernandez Perez Tomei, E. M. Gregores, D. S. Lemos, P. G. Mercadante, S. F. Novaes, Sandra S. Padula, A. Aleksandrov, G. Antchev, I. Atanasov, R. Hadjiiska, P. Iaydjiev, M. Misheva, M. Rodozov, M. Shopova, G. Sultanov, M. Bonchev, A. Dimitrov, T. Ivanov, L. Litov, B. Pavlov, P. Petkov, A. Petrov, W. Fang, Q. Guo, H. Wang, L. Yuan, M. Ahmad, Z. Hu, Y. Wang, K. Yi, E. Chapon, G. M. Chen, H. S. Chen, M. Chen, T. Javaid, A. Kapoor, D. Leggat, H. Liao, Z. Liu, R. Sharma, A. Spiezia, J. Tao, J. Thomas-wilsker, J. Wang, H. Zhang, S. Zhang, J. Zhao, A. Agapitos, Y. Ban, C. Chen, Q. Huang, A. Levin, Q. Li, M. Lu, X. Lyu, Y. Mao, S. J. Qian, D. Wang, Q. Wang, J. Xiao, Z. You, X. Gao, M. Xiao, C. Avila, A. Cabrera, C. Florez, J. Fraga, A. Sarkar, M. A. Segura Delgado, J. Jaramillo, J. Mejia Guisao, F. Ramirez, J. D. Ruiz Alvarez, C. A. Salazar González, N. Vanegas Arbelaez, D. Giljanovic, N. Godinovic, D. Lelas, I. Puljak, Z. Antunovic, M. Kovac, T. Sculac, V. Brigljevic, D. Ferencek, D. Majumder, M. Roguljic, A. Starodumov, T. Susa, M. W. Ather, A. Attikis, E. Erodotou, A. Ioannou, G. Kole, M. Kolosova, S. Konstantinou, J. Mousa, C. Nicolaou, F. Ptochos, P. A. Razis, H. Rykaczewski, H. Saka, D. Tsiakkouri, M. Finger, M. Finger Jr., A. Kveton, J. Tomsa, E. Ayala, E. Carrera Jarrin, H. Abdalla, Y. Assran, S. Khalil, A. Lotfy, M. A. Mahmoud, S. Bhowmik, A. Carvalho Antunes De Oliveira, R. K. Dewanjee, K. Ehataht, M. Kadastik, M. Raidal, C. Veelken, P. Eerola, L. Forthomme, H. Kirschenmann, K. Osterberg, M. Voutilainen, E. Brücken, F. Garcia, J. Havukainen, V. Karimäki, M. S. Kim, R. Kinnunen, T. Lampén, K. Lassila-Perini, S. Lehti, T. Lindén, H. Siikonen, E. Tuominen, J. Tuominiemi, P. Luukka, T. Tuuva, C. Amendola, M. Besancon, F. Couderc, M. Dejardin, D. Denegri, J. L. Faure, F. Ferri, S. Ganjour, A. Givernaud, P. Gras, G. Hamel de Monchenault, P. Jarry, B. Lenzi, E. Locci, J. Malcles, J. Rander, A. Rosowsky, M. Ö. Sahin, A. Savoy-Navarro, M. Titov, G. B. Yu, S. Ahuja, F. Beaudette, M. Bonanomi, A. Buchot Perraguin, P. Busson, C. Charlot, O. Davignon, B. Diab, G. Falmagne, R. Granier de Cassagnac, A. Hakimi, I. Kucher, A. Lobanov, C. Martin Perez, M. Nguyen, C. Ochando, P. Paganini, J. Rembser, R. Salerno, J. B. Sauvan, Y. Sirois, A. Zabi, A. Zghiche, J.-L. Agram, J. Andrea, D. Bloch, G. Bourgatte, J.-M. Brom, E. C. Chabert, C. Collard, J.-C. Fontaine, D. Gelé, U. Goerlach, C. Grimault, A.-C. Le Bihan, P. Van Hove, E. Asilar, S. Beauceron, C. Bernet, G. Boudoul, C. Camen, A. Carle, N. Chanon, D. Contardo, P. Depasse, H. El Mamouni, J. Fay, S. Gascon, M. Gouzevitch, B. Ille, Sa. Jain, I. B. Laktineh, H. Lattaud, A. Lesauvage, M. Lethuillier, L. Mirabito, L. Torterotot, G. Touquet, M. Vander Donckt, S. Viret, A. Khvedelidze, Z. Tsamalaidze, L. Feld, K. Klein, M. Lipinski, D. Meuser, A. Pauls, M. Preuten, M. P. Rauch, J. Schulz, M. Teroerde, D. Eliseev, M. Erdmann, P. Fackeldey, B. Fischer, S. Ghosh, T. Hebbeker, K. Hoepfner, H. Keller, L. Mastrolorenzo, M. Merschmeyer, A. Meyer, G. Mocellin, S. Mondal, S. Mukherjee, D. Noll, A. Novak, T. Pook, A. Pozdnyakov, Y. Rath, H. Reithler, J. Roemer, A. Schmidt, S. C. Schuler, A. Sharma, S. Wiedenbeck, S. Zaleski, C. Dziwok, G. Flügge, W. Haj Ahmad, O. Hlushchenko, T. Kress, A. Nowack, C. Pistone, O. Pooth, D. Roy, H. Sert, A. Stahl, T. Ziemons, H. Aarup Petersen, M. Aldaya Martin, P. Asmuss, I. Babounikau, S. Baxter, O. Behnke, A. Bermúdez Martínez, A. A. Bin Anuar, K. Borras, V. Botta, D. Brunner, A. Campbell, A. Cardini, P. Connor, S. Consuegra Rodríguez, V. Danilov, A. De Wit, M. M. Defranchis, L. Didukh, D. Domínguez Damiani, G. Eckerlin, D. Eckstein, T. Eichhorn, L. I. Estevez Banos, E. Gallo, A. Geiser, A. Giraldi, A. Grohsjean, M. Guthoff, A. Harb, A. Jafari, N. Z. Jomhari, H. Jung, A. Kasem, M. Kasemann, H. Kaveh, C. Kleinwort, J. Knolle, D. Krücker, W. Lange, T. Lenz, J. Lidrych, K. Lipka, W. Lohmann, R. Mankel, I.-A. Melzer-Pellmann, J. Metwally, A. B. Meyer, M. Meyer, M. Missiroli, J. Mnich, A. Mussgiller, V. Myronenko, Y. Otarid, D. Pérez Adán, S. K. Pflitsch, D. Pitzl, A. Raspereza, A. Saggio, A. Saibel, M. Savitskyi, V. Scheurer, C. Schwanenberger, A. Singh, R. E. Sosa Ricardo, N. Tonon, O. Turkot, A. Vagnerini, M. Van De Klundert, R. Walsh, D. Walter, Y. Wen, K. Wichmann, C. Wissing, S. Wuchterl, O. Zenaiev, R. Zlebcik, R. Aggleton, S. Bein, L. Benato, A. Benecke, K. De Leo, T. Dreyer, A. Ebrahimi, M. Eich, F. Feindt, A. Fröhlich, C. Garbers, E. Garutti, P. Gunnellini, J. Haller, A. Hinzmann, A. Karavdina, G. Kasieczka, R. Klanner, R. Kogler, V. Kutzner, J. Lange, T. Lange, A. Malara, C. E. N. Niemeyer, A. Nigamova, K. J. Pena Rodriguez, O. Rieger, P. Schleper, S. Schumann, J. Schwandt, D. Schwarz, J. Sonneveld, H. Stadie, G. Steinbrück, B. Vormwald, I. Zoi, J. Bechtel, T. Berger, E. Butz, R. Caspart, T. Chwalek, W. De Boer, A. Dierlamm, A. Droll, K. El Morabit, N. Faltermann, K. Flöh, M. Giffels, A. Gottmann, F. Hartmann, C. Heidecker, U. Husemann, M. A. Iqbal, I. Katkov, P. Keicher, R. Koppenhöfer, S. Maier, M. Metzler, S. Mitra, D. Müller, Th. Müller, M. Musich, G. Quast, K. Rabbertz, J. Rauser, D. Savoiu, D. Schäfer, M. Schnepf, M. Schröder, D. Seith, I. Shvetsov, H. J. Simonis, R. Ulrich, M. Wassmer, M. Weber, R. Wolf, S. Wozniewski, G. Anagnostou, P. Asenov, G. Daskalakis, T. Geralis, A. Kyriakis, D. Loukas, G. Paspalaki, A. Stakia, M. Diamantopoulou, D. Karasavvas, G. Karathanasis, P. Kontaxakis, C. K. Koraka, A. Manousakis-katsikakis, A. Panagiotou, I. Papavergou, N. Saoulidou, K. Theofilatos, K. Vellidis, E. Vourliotis, G. Bakas, K. Kousouris, I. Papakrivopoulos, G. Tsipolitis, A. Zacharopoulou, I. Evangelou, C. Foudas, P. Gianneios, P. Katsoulis, P. Kokkas, K. Manitara, N. Manthos, I. Papadopoulos, J. Strologas, M. Bartók, M. Csanad, M. M. A. Gadallah, S. Lökös, P. Major, K. Mandal, A. Mehta, G. Pasztor, O. Surányi, G. I. Veres, G. Bencze, C. Hajdu, D. Horvath, F. Sikler, V. Veszpremi, G. Vesztergombi, S. Czellar, J. Karancsi, J. Molnar, Z. Szillasi, D. Teyssier, P. Raics, Z. L. Trocsanyi, B. Ujvari, T. Csorgo, F. Nemes, T. Novak, S. Choudhury, J. R. Komaragiri, D. Kumar, L. Panwar, P. C. Tiwari, S. Bahinipati, D. Dash, C. Kar, P. Mal, T. Mishra, V. K. Muraleedharan Nair Bindhu, A. Nayak, D. K. Sahoo, N. Sur, S. K. Swain, S. Bansal, S. B. Beri, V. Bhatnagar, G. Chaudhary, S. Chauhan, N. Dhingra, R. Gupta, A. Kaur, S. Kaur, P. Kumari, M. Meena, K. Sandeep, S. Sharma, J. B. Singh, A. K. Virdi, A. Ahmed, A. Bhardwaj, B. C. Choudhary, R. B. Garg, M. Gola, S. Keshri, A. Kumar, M. Naimuddin, P. Priyanka, K. Ranjan, A. Shah, M. Bharti, R. Bhattacharya, S. Bhattacharya, D. Bhowmik, S. Dutta, S. Ghosh, B. Gomber, M. Maity, S. Nandan, P. Palit, P. K. Rout, G. Saha, B. Sahu, S. Sarkar, M. Sharan, B. Singh, S. Thakur, P. K. Behera, S. C. Behera, P. Kalbhor, A. Muhammad, R. Pradhan, P. R. Pujahari, A. Sharma, A. K. Sikdar, D. Dutta, V. Kumar, K. Naskar, P. K. Netrakanti, L. M. Pant, P. Shukla, T. Aziz, M. A. Bhat, S. Dugad, R. Kumar Verma, G. B. Mohanty, U. Sarkar, S. Banerjee, S. Bhattacharya, S. Chatterjee, R. Chudasama, M. Guchait, S. Karmakar, S. Kumar, G. Majumder, K. Mazumdar, S. Mukherjee, D. Roy, S. Dube, B. Kansal, S. Pandey, A. Rane, A. Rastogi, S. Sharma, H. Bakhshiansohi, M. Zeinali, S. Chenarani, S. M. Etesami, M. Khakzad, M. Mohammadi Najafabadi, M. Felcini, M. Grunewald, M. Abbrescia, R. Aly, C. Aruta, A. Colaleo, D. Creanza, N. De Filippis, M. De Palma, A. Di Florio, A. Di Pilato, W. Elmetenawee, L. Fiore, A. Gelmi, M. Gul, G. Iaselli, M. Ince, S. Lezki, G. Maggi, M. Maggi, I. Margjeka, V. Mastrapasqua, J. A. Merlin, S. My, S. Nuzzo, A. Pompili, G. Pugliese, A. Ranieri, G. Selvaggi, L. Silvestris, F. M. Simone, R. Venditti, P. Verwilligen, G. Abbiendi, C. Battilana, D. Bonacorsi, L. Borgonovi, S. Braibant-Giacomelli, R. Campanini, P. Capiluppi, A. Castro, F. R. Cavallo, C. Ciocca, M. Cuffiani, G. M. Dallavalle, T. Diotalevi, F. Fabbri, A. Fanfani, E. Fontanesi, P. Giacomelli, L. Giommi, C. Grandi, L. Guiducci, F. Iemmi, S. Lo Meo, S. Marcellini, G. Masetti, F. L. Navarria, A. Perrotta, F. Primavera, A. M. Rossi, T. Rovelli, G. P. Siroli, N. Tosi, S. Albergo, S. Costa, A. Di Mattia, R. Potenza, A. Tricomi, C. Tuve, G. Barbagli, A. Cassese, R. Ceccarelli, V. Ciulli, C. Civinini, R. D’Alessandro, F. Fiori, E. Focardi, G. Latino, P. Lenzi, M. Lizzo, M. Meschini, S. Paoletti, R. Seidita, G. Sguazzoni, L. Viliani, L. Benussi, S. Bianco, D. Piccolo, M. Bozzo, F. Ferro, R. Mulargia, E. Robutti, S. Tosi, A. Benaglia, A. Beschi, F. Brivio, F. Cetorelli, V. Ciriolo, F. De Guio, M. E. Dinardo, P. Dini, S. Gennai, A. Ghezzi, P. Govoni, L. Guzzi, M. Malberti, S. Malvezzi, A. Massironi, D. Menasce, F. Monti, L. Moroni, M. Paganoni, D. Pedrini, S. Ragazzi, T. Tabarelli de Fatis, D. Valsecchi, D. Zuolo, S. Buontempo, N. Cavallo, A. De Iorio, F. Fabozzi, F. Fienga, A. O. M. Iorio, L. Lista, S. Meola, P. Paolucci, B. Rossi, C. Sciacca, E. Voevodina, P. Azzi, N. Bacchetta, D. Bisello, P. Bortignon, A. Bragagnolo, R. Carlin, P. Checchia, P. De Castro Manzano, T. Dorigo, F. Gasparini, U. Gasparini, S. Y. Hoh, L. Layer, M. Margoni, A. T. Meneguzzo, M. Presilla, P. Ronchese, R. Rossin, F. Simonetto, G. Strong, M. Tosi, H. YARAR, M. Zanetti, P. Zotto, A. Zucchetta, G. Zumerle, C. Aimè, A. Braghieri, S. Calzaferri, D. Fiorina, P. Montagna, S. P. Ratti, V. Re, M. Ressegotti, C. Riccardi, P. Salvini, I. Vai, P. Vitulo, M. Biasini, G. M. Bilei, D. Ciangottini, L. Fanò, P. Lariccia, G. Mantovani, V. Mariani, M. Menichelli, F. Moscatelli, A. Piccinelli, A. Rossi, A. Santocchia, D. Spiga, T. Tedeschi, K. Androsov, P. Azzurri, G. Bagliesi, V. Bertacchi, L. Bianchini, T. Boccali, R. Castaldi, M. A. Ciocci, R. Dell’Orso, M. R. Di Domenico, S. Donato, L. Giannini, A. Giassi, M. T. Grippo, F. Ligabue, E. Manca, G. Mandorli, A. Messineo, F. Palla, G. Ramirez-Sanchez, A. Rizzi, G. Rolandi, S. Roy Chowdhury, A. Scribano, N. Shafiei, P. Spagnolo, R. Tenchini, G. Tonelli, N. Turini, A. Venturi, P. G. Verdini, F. Cavallari, M. Cipriani, D. Del Re, E. Di Marco, M. Diemoz, E. Longo, P. Meridiani, G. Organtini, F. Pandolfi, R. Paramatti, C. Quaranta, S. Rahatlou, C. Rovelli, F. Santanastasio, L. Soffi, R. Tramontano, N. Amapane, R. Arcidiacono, S. Argiro, M. Arneodo, N. Bartosik, R. Bellan, A. Bellora, J. Berenguer Antequera, C. Biino, A. Cappati, N. Cartiglia, S. Cometti, M. Costa, R. Covarelli, N. Demaria, B. Kiani, F. Legger, C. Mariotti, S. Maselli, E. Migliore, V. Monaco, E. Monteil, M. Monteno, M. M. Obertino, G. Ortona, L. Pacher, N. Pastrone, M. Pelliccioni, G. L. Pinna Angioni, M. Ruspa, R. Salvatico, F. Siviero, V. Sola, A. Solano, D. Soldi, A. Staiano, M. Tornago, D. Trocino, S. Belforte, V. Candelise, M. Casarsa, F. Cossutti, A. Da Rold, G. Della Ricca, F. Vazzoler, S. Dogra, C. Huh, B. Kim, D. H. Kim, G. N. Kim, J. Lee, S. W. Lee, C. S. Moon, Y. D. Oh, S. I. Pak, B. C. Radburn-Smith, S. Sekmen, Y. C. Yang, H. Kim, D. H. Moon, B. Francois, T. J. Kim, J. Park, S. Cho, S. Choi, Y. Go, S. Ha, B. Hong, K. Lee, K. S. Lee, J. Lim, J. Park, S. K. Park, J. Yoo, J. Goh, A. Gurtu, H. S. Kim, Y. Kim, J. Almond, J. H. Bhyun, J. Choi, S. Jeon, J. Kim, J. S. Kim, S. Ko, H. Kwon, H. Lee, K. Lee, S. Lee, K. Nam, B. H. Oh, M. Oh, S. B. Oh, H. Seo, U. K. Yang, I. Yoon, D. Jeon, J. H. Kim, B. Ko, J. S. H. Lee, I. C. Park, Y. Roh, D. Song, I. J. Watson, H. D. Yoo, Y. Choi, C. Hwang, Y. Jeong, H. Lee, Y. Lee, I. Yu, Y. Maghrbi, V. Veckalns, A. Juodagalvis, A. Rinkevicius, G. Tamulaitis, A. Vaitkevicius, W. A. T. Wan Abdullah, M. N. Yusli, Z. Zolkapli, J. F. Benitez, A. Castaneda Hernandez, J. A. Murillo Quijada, L. Valencia Palomo, G. Ayala, H. Castilla-Valdez, E. De La Cruz-Burelo, I. Heredia-De La Cruz, R. Lopez-Fernandez, C. A. Mondragon Herrera, D. A. Perez Navarro, A. Sanchez-Hernandez, S. Carrillo Moreno, C. Oropeza Barrera, M. Ramirez-Garcia, F. Vazquez Valencia, J. Eysermans, I. Pedraza, H. A. Salazar Ibarguen, C. Uribe Estrada, A. Morelos Pineda, J. Mijuskovic, N. Raicevic, D. Krofcheck, S. Bheesette, P. H. Butler, A. Ahmad, M. I. Asghar, A. Awais, M. I. M. Awan, H. R. Hoorani, W. A. Khan, M. A. Shah, M. Shoaib, M. Waqas, V. Avati, L. Grzanka, M. Malawski, H. Bialkowska, M. Bluj, B. Boimska, T. Frueboes, M. Górski, M. Kazana, M. Szleper, P. Traczyk, P. Zalewski, K. Bunkowski, K. Doroba, A. Kalinowski, M. Konecki, J. Krolikowski, M. Walczak, M. Araujo, P. Bargassa, D. Bastos, A. Boletti, P. Faccioli, M. Gallinaro, J. Hollar, N. Leonardo, T. Niknejad, J. Seixas, K. Shchelina, O. Toldaiev, J. Varela, A. Baginyan, P. Bunin, Y. Ershov, A. Golunov, I. Golutvin, N. Gorbounov, I. Gorbunov, A. Kamenev, V. Karjavine, A. Lanev, A. Malakhov, V. Matveev, V. Palichik, V. Perelygin, M. Savina, V. Shalaev, S. Shmatov, O. Teryaev, N. Voytishin, B. S. Yuldashev, A. Zarubin, I. Zhizhin, G. Gavrilov, V. Golovtcov, Y. Ivanov, V. Kim, E. Kuznetsova, V. Murzin, V. Oreshkin, I. Smirnov, D. Sosnov, V. Sulimov, L. Uvarov, S. Volkov, A. Vorobyev, Yu. Andreev, A. Dermenev, S. Gninenko, N. Golubev, A. Karneyeu, M. Kirsanov, N. Krasnikov, A. Pashenkov, G. Pivovarov, D. Tlisov, A. Toropin, V. Epshteyn, V. Gavrilov, N. Lychkovskaya, A. Nikitenko, V. Popov, G. Safronov, A. Spiridonov, A. Stepennov, M. Toms, E. Vlasov, A. Zhokin, T. Aushev, R. Chistov, M. Danilov, A. Oskin, P. Parygin, S. Polikarpov, V. Andreev, M. Azarkin, I. Dremin, M. Kirakosyan, A. Terkulov, A. Belyaev, E. Boos, V. Bunichev, M. Dubinin, L. Dudko, A. Gribushin, V. Klyukhin, O. Kodolova, I. Lokhtin, S. Obraztsov, M. Perfilov, S. Petrushanko, V. Savrin, V. Blinov, T. Dimova, L. Kardapoltsev, I. Ovtin, Y. Skovpen, I. Azhgirey, I. Bayshev, V. Kachanov, A. Kalinin, D. Konstantinov, V. Petrov, R. Ryutin, A. Sobol, S. Troshin, N. Tyurin, A. Uzunian, A. Volkov, A. Babaev, A. Iuzhakov, V. Okhotnikov, L. Sukhikh, V. Borchsh, V. Ivanchenko, E. Tcherniaev, P. Adzic, P. Cirkovic, M. Dordevic, P. Milenovic, J. Milosevic, M. Aguilar-Benitez, J. Alcaraz Maestre, A. Álvarez Fernández, I. Bachiller, M. Barrio Luna, Cristina F. Bedoya, J. A. Brochero Cifuentes, C. A. Carrillo Montoya, M. Cepeda, M. Cerrada, N. Colino, B. De La Cruz, A. Delgado Peris, J. P. Fernández Ramos, J. Flix, M. C. Fouz, A. García Alonso, O. Gonzalez Lopez, S. Goy Lopez, J. M. Hernandez, M. I. Josa, J. León Holgado, D. Moran, Á. Navarro Tobar, A. Pérez-Calero Yzquierdo, J. Puerta Pelayo, I. Redondo, L. Romero, S. Sánchez Navas, M. S. Soares, A. Triossi, L. Urda Gómez, C. Willmott, C. Albajar, J. F. de Trocóniz, R. Reyes-Almanza, B. Alvarez Gonzalez, J. Cuevas, C. Erice, J. Fernandez Menendez, S. Folgueras, I. Gonzalez Caballero, E. Palencia Cortezon, C. Ramón Álvarez, J. Ripoll Sau, V. Rodríguez Bouza, S. Sanchez Cruz, A. Trapote, I. J. Cabrillo, A. Calderon, B. Chazin Quero, J. Duarte Campderros, M. Fernandez, P. J. Fernández Manteca, G. Gomez, C. Martinez Rivero, P. Martinez Ruiz del Arbol, F. Matorras, J. Piedra Gomez, C. Prieels, F. Ricci-Tam, T. Rodrigo, A. Ruiz-Jimeno, L. Scodellaro, I. Vila, J. M. Vizan Garcia, M K Jayananda, B. Kailasapathy, D. U. J. Sonnadara, DDC Wickramarathna, W. G. D. Dharmaratna, K. Liyanage, N. Perera, N. Wickramage, T. K. Aarrestad, D. Abbaneo, B. Akgun, E. Auffray, G. Auzinger, J. Baechler, P. Baillon, A. H. Ball, D. Barney, J. Bendavid, N. Beni, M. Bianco, A. Bocci, E. Bossini, E. Brondolin, T. Camporesi, M. Capeans Garrido, G. Cerminara, L. Cristella, D. d’Enterria, A. Dabrowski, N. Daci, V. Daponte, A. David, A. De Roeck, M. Deile, R. Di Maria, M. Dobson, M. Dünser, N. Dupont, A. Elliott-Peisert, N. Emriskova, F. Fallavollita, D. Fasanella, S. Fiorendi, A. Florent, G. Franzoni, J. Fulcher, W. Funk, S. Giani, D. Gigi, K. Gill, F. Glege, L. Gouskos, M. Guilbaud, D. Gulhan, M. Haranko, J. Hegeman, Y. Iiyama, V. Innocente, T. James, P. Janot, J. Kaspar, J. Kieseler, M. Komm, N. Kratochwil, C. Lange, S. Laurila, P. Lecoq, K. Long, C. Lourenço, L. Malgeri, S. Mallios, M. Mannelli, F. Meijers, S. Mersi, E. Meschi, F. Moortgat, M. Mulders, J. Niedziela, S. Orfanelli, L. Orsini, F. Pantaleo, L. Pape, E. Perez, M. Peruzzi, A. Petrilli, G. Petrucciani, A. Pfeiffer, M. Pierini, T. Quast, D. Rabady, A. Racz, M. Rieger, M. Rovere, H. Sakulin, J. Salfeld-Nebgen, S. Scarfi, C. Schäfer, C. Schwick, M. Selvaggi, A. Sharma, P. Silva, W. Snoeys, P. Sphicas, S. Summers, V. R. Tavolaro, D. Treille, A. Tsirou, G. P. Van Onsem, A. Vartak, M. Verzetti, K. A. Wozniak, W. D. Zeuner, L. Caminada, W. Erdmann, R. Horisberger, Q. Ingram, H. C. Kaestli, D. Kotlinski, U. Langenegger, T. Rohe, M. Backhaus, P. Berger, A. Calandri, N. Chernyavskaya, A. De Cosa, G. Dissertori, M. Dittmar, M. Donegà, C. Dorfer, T. Gadek, T. A. Gómez Espinosa, C. Grab, D. Hits, W. Lustermann, A.-M. Lyon, R. A. Manzoni, M. T. Meinhard, F. Micheli, F. Nessi-Tedaldi, F. Pauss, V. Perovic, G. Perrin, S. Pigazzini, M. G. Ratti, M. Reichmann, C. Reissel, T. Reitenspiess, B. Ristic, D. Ruini, D. A. Sanz Becerra, M. Schönenberger, V. Stampf, J. Steggemann, M. L. Vesterbacka Olsson, R. Wallny, D. H. Zhu, C. Amsler, C. Botta, D. Brzhechko, M. F. Canelli, R. Del Burgo, J. K. Heikkilä, M. Huwiler, A. Jofrehei, B. Kilminster, S. Leontsinis, A. Macchiolo, P. Meiring, V. M. Mikuni, U. Molinatti, I. Neutelings, G. Rauco, A. Reimers, P. Robmann, K. Schweiger, Y. Takahashi, C. Adloff, C. M. Kuo, W. Lin, A. Roy, T. Sarkar, S. S. Yu, L. Ceard, P. Chang, Y. Chao, K. F. Chen, P. H. Chen, W.-S. Hou, Y. y. Li, R.-S. Lu, E. Paganis, A. Psallidas, A. Steen, E. Yazgan, B. Asavapibhop, C. Asawatangtrakuldee, N. Srimanobhas, F. Boran, S. Damarseckin, Z. S. Demiroglu, F. Dolek, C. Dozen, I. Dumanoglu, E. Eskut, G. Gokbulut, Y. Guler, E. Gurpinar Guler, I. Hos, C. Isik, E. E. Kangal, O. Kara, A. Kayis Topaksu, U. Kiminsu, G. Onengut, K. Ozdemir, A. Polatoz, A. E. Simsek, B. Tali, U. G. Tok, S. Turkcapar, I. S. Zorbakir, C. Zorbilmez, B. Isildak, G. Karapinar, K. Ocalan, M. Yalvac, I. O. Atakisi, E. Gülmez, M. Kaya, O. Kaya, Ö. Özçelik, S. Tekten, E. A. Yetkin, A. Cakir, K. Cankocak, Y. Komurcu, S. Sen, F. Aydogmus Sen, S. Cerci, B. Kaynak, S. Ozkorucuklu, D. Sunar Cerci, B. Grynyov, L. Levchuk, E. Bhal, S. Bologna, J. J. Brooke, E. Clement, D. Cussans, H. Flacher, J. Goldstein, G. P. Heath, H. F. Heath, L. Kreczko, B. Krikler, S. Paramesvaran, T. Sakuma, S. Seif El Nasr-Storey, V. J. Smith, N. Stylianou, J. Taylor, A. Titterton, K. W. Bell, A. Belyaev, C. Brew, R. M. Brown, D. J. A. Cockerill, K. V. Ellis, K. Harder, S. Harper, J. Linacre, K. Manolopoulos, D. M. Newbold, E. Olaiya, D. Petyt, T. Reis, T. Schuh, C. H. Shepherd-Themistocleous, A. Thea, I. R. Tomalin, T. Williams, R. Bainbridge, P. Bloch, S. Bonomally, J. Borg, S. Breeze, O. Buchmuller, A. Bundock, V. Cepaitis, G. S. Chahal, D. Colling, P. Dauncey, G. Davies, M. Della Negra, G. Fedi, G. Hall, G. Iles, J. Langford, L. Lyons, A.-M. Magnan, S. Malik, A. Martelli, V. Milosevic, J. Nash, V. Palladino, M. Pesaresi, D. M. Raymond, A. Richards, A. Rose, E. Scott, C. Seez, A. Shtipliyski, M. Stoye, A. Tapper, K. Uchida, T. Virdee, N. Wardle, S. N. Webb, D. Winterbottom, A. G. Zecchinelli, J. E. Cole, P. R. Hobson, A. Khan, P. Kyberd, C. K. Mackay, I. D. Reid, L. Teodorescu, S. Zahid, S. Abdullin, A. Brinkerhoff, K. Call, B. Caraway, J. Dittmann, K. Hatakeyama, A. R. Kanuganti, C. Madrid, B. McMaster, N. Pastika, S. Sawant, C. Smith, J. Wilson, R. Bartek, A. Dominguez, R. Uniyal, A. M. Vargas Hernandez, A. Buccilli, O. Charaf, S. I. Cooper, S. V. Gleyzer, C. Henderson, P. Rumerio, C. West, A. Akpinar, A. Albert, D. Arcaro, C. Cosby, Z. Demiragli, D. Gastler, J. Rohlf, K. Salyer, D. Sperka, D. Spitzbart, I. Suarez, S. Yuan, D. Zou, G. Benelli, B. Burkle, X. Coubez, D. Cutts, Y. t. Duh, M. Hadley, U. Heintz, J. M. Hogan, K. H. M. Kwok, E. Laird, G. Landsberg, K. T. Lau, J. Lee, M. Narain, S. Sagir, R. Syarif, E. Usai, W. Y. Wong, D. Yu, W. Zhang, R. Band, C. Brainerd, R. Breedon, M. Calderon De La BarcaSanchez, M. Chertok, J. Conway, R. Conway, P. T. Cox, R. Erbacher, C. Flores, G. Funk, F. Jensen, W. Ko, O. Kukral, R. Lander, M. Mulhearn, D. Pellett, J. Pilot, M. Shi, D. Taylor, K. Tos, M. Tripathi, Y. Yao, F. Zhang, M. Bachtis, R. Cousins, A. Dasgupta, D. Hamilton, J. Hauser, M. Ignatenko, T. Lam, N. Mccoll, W. A. Nash, S. Regnard, D. Saltzberg, C. Schnaible, B. Stone, V. Valuev, K. Burt, Y. Chen, R. Clare, J. W. Gary, G. Hanson, G. Karapostoli, O. R. Long, N. Manganelli, M. Olmedo Negrete, M. I. Paneva, W. Si, S. Wimpenny, Y. Zhang, J. G. Branson, P. Chang, S. Cittolin, S. Cooperstein, N. Deelen, J. Duarte, R. Gerosa, D. Gilbert, V. Krutelyov, J. Letts, M. Masciovecchio, S. May, S. Padhi, M. Pieri, V. Sharma, M. Tadel, F. Würthwein, A. Yagil, N. Amin, C. Campagnari, M. Citron, A. Dorsett, V. Dutta, J. Incandela, B. Marsh, H. Mei, A. Ovcharova, H. Qu, M. Quinnan, J. Richman, U. Sarica, D. Stuart, S. Wang, A. Bornheim, O. Cerri, I. Dutta, J. M. Lawhorn, N. Lu, J. Mao, H.B. Newman, J. Ngadiuba, T. Q. Nguyen, J. Pata, M. Spiropulu, J. R. Vlimant, C. Wang, S. Xie, Z. Zhang, R. Y. Zhu, J. Alison, M. B. Andrews, T. Ferguson, T. Mudholkar, M. Paulini, M. Sun, I. Vorobiev, J. P. Cumalat, W. T. Ford, E. MacDonald, T. Mulholland, R. Patel, A. Perloff, K. Stenson, K. A. Ulmer, S. R. Wagner, J. Alexander, Y. Cheng, J. Chu, D. J. Cranshaw, A. Datta, A. Frankenthal, K. Mcdermott, J. Monroy, J. R. Patterson, D. Quach, A. Ryd, W. Sun, S. M. Tan, Z. Tao, J. Thom, P. Wittich, M. Zientek, M. Albrow, M. Alyari, G. Apollinari, A. Apresyan, A. Apyan, S. Banerjee, L. A. T. Bauerdick, A. Beretvas, D. Berry, J. Berryhill, P. C. Bhat, K. Burkett, J. N. Butler, A. Canepa, G. B. Cerati, H. W. K. Cheung, F. Chlebana, M. Cremonesi, V. D. Elvira, J. Freeman, Z. Gecse, E. Gottschalk, L. Gray, D. Green, S. Grünendahl, O. Gutsche, R. M. Harris, S. Hasegawa, R. Heller, T. C. Herwig, J. Hirschauer, B. Jayatilaka, S. Jindariani, M. Johnson, U. Joshi, P. Klabbers, T. Klijnsma, B. Klima, M. J. Kortelainen, S. Lammel, D. Lincoln, R. Lipton, M. Liu, T. Liu, J. Lykken, K. Maeshima, D. Mason, P. McBride, P. Merkel, S. Mrenna, S. Nahn, V. O’Dell, V. Papadimitriou, K. Pedro, C. Pena, O. Prokofyev, F. Ravera, A. Reinsvold Hall, L. Ristori, B. Schneider, E. Sexton-Kennedy, N. Smith, A. Soha, W. J. Spalding, L. Spiegel, S. Stoynev, J. Strait, L. Taylor, S. Tkaczyk, N. V. Tran, L. Uplegger, E. W. Vaandering, H. A. Weber, A. Woodard, D. Acosta, P. Avery, D. Bourilkov, L. Cadamuro, V. Cherepanov, F. Errico, R. D. Field, D. Guerrero, B. M. Joshi, M. Kim, J. Konigsberg, A. Korytov, K. H. Lo, K. Matchev, N. Menendez, G. Mitselmakher, D. Rosenzweig, K. Shi, J. Sturdy, J. Wang, S. Wang, X. Zuo, T. Adams, A. Askew, D. Diaz, R. Habibullah, S. Hagopian, V. Hagopian, K. F. Johnson, R. Khurana, T. Kolberg, G. Martinez, H. Prosper, C. Schiber, R. Yohay, J. Zhang, M. M. Baarmand, S. Butalla, T. Elkafrawy, M. Hohlmann, D. Noonan, M. Rahmani, M. Saunders, F. Yumiceva, M. R. Adams, L. Apanasevich, H. Becerril Gonzalez, R. Cavanaugh, X. Chen, S. Dittmer, O. Evdokimov, C. E. Gerber, D. A. Hangal, D. J. Hofman, C. Mills, G. Oh, T. Roy, M. B. Tonjes, N. Varelas, J. Viinikainen, X. Wang, Z. Wu, Z. Ye, M. Alhusseini, K. Dilsiz, S. Durgut, R. P. Gandrajula, M. Haytmyradov, V. Khristenko, O. K. Köseyan, J.-P. Merlo, A. Mestvirishvili, A. Moeller, J. Nachtman, H. Ogul, Y. Onel, F. Ozok, A. Penzo, C. Snyder, E. Tiras, J. Wetzel, O. Amram, B. Blumenfeld, L. Corcodilos, M. Eminizer, A. V. Gritsan, S. Kyriacou, P. Maksimovic, C. Mantilla, J. Roskes, M. Swartz, T. Á. Vámi, C. Baldenegro Barrera, P. Baringer, A. Bean, A. Bylinkin, T. Isidori, S. Khalil, J. King, G. Krintiras, A. Kropivnitskaya, C. Lindsey, N. Minafra, M. Murray, C. Rogan, C. Royon, S. Sanders, E. Schmitz, J. D. Tapia Takaki, Q. Wang, J. Williams, G. Wilson, S. Duric, A. Ivanov, K. Kaadze, D. Kim, Y. Maravin, T. Mitchell, A. Modak, A. Mohammadi, F. Rebassoo, D. Wright, E. Adams, A. Baden, O. Baron, A. Belloni, S. C. Eno, Y. Feng, N. J. Hadley, S. Jabeen, G. Y. Jeng, R. G. Kellogg, T. Koeth, A. C. Mignerey, S. Nabili, M. Seidel, A. Skuja, S. C. Tonwar, L. Wang, K. Wong, D. Abercrombie, B. Allen, R. Bi, S. Brandt, W. Busza, I. A. Cali, Y. Chen, M. D’Alfonso, G. Gomez Ceballos, M. Goncharov, P. Harris, D. Hsu, M. Hu, M. Klute, D. Kovalskyi, J. Krupa, Y.-J. Lee, P. D. Luckey, B. Maier, A. C. Marini, C. Mcginn, C. Mironov, S. Narayanan, X. Niu, C. Paus, D. Rankin, C. Roland, G. Roland, Z. Shi, G. S. F. Stephans, K. Sumorok, K. Tatar, D. Velicanu, J. Wang, T. W. Wang, Z. Wang, B. Wyslouch, R. M. Chatterjee, A. Evans, P. Hansen, J. Hiltbrand, Sh. Jain, M. Krohn, Y. Kubota, Z. Lesko, J. Mans, M. Revering, R. Rusack, R. Saradhy, N. Schroeder, N. Strobbe, M. A. Wadud, J. G. Acosta, S. Oliveros, K. Bloom, S. Chauhan, D. R. Claes, C. Fangmeier, L. Finco, F. Golf, J. R. González Fernández, I. Kravchenko, J. E. Siado, G. R. Snow, W. Tabb, F. Yan, G. Agarwal, H. Bandyopadhyay, C. Harrington, L. Hay, I. Iashvili, A. Kharchilava, C. McLean, D. Nguyen, J. Pekkanen, S. Rappoccio, B. Roozbahani, G. Alverson, E. Barberis, C. Freer, Y. Haddad, A. Hortiangtham, J. Li, G. Madigan, B. Marzocchi, D. M. Morse, V. Nguyen, T. Orimoto, A. Parker, L. Skinnari, A. Tishelman-Charny, T. Wamorkar, B. Wang, A. Wisecarver, D. Wood, S. Bhattacharya, J. Bueghly, Z. Chen, A. Gilbert, T. Gunter, K. A. Hahn, N. Odell, M. H. Schmitt, K. Sung, M. Velasco, R. Bucci, N. Dev, R. Goldouzian, M. Hildreth, K. Hurtado Anampa, C. Jessop, D. J. Karmgard, K. Lannon, N. Loukas, N. Marinelli, I. Mcalister, F. Meng, K. Mohrman, Y. Musienko, R. Ruchti, P. Siddireddy, S. Taroni, M. Wayne, A. Wightman, M. Wolf, L. Zygala, J. Alimena, B. Bylsma, B. Cardwell, L. S. Durkin, B. Francis, C. Hill, A. Lefeld, B. L. Winer, B. R. Yates, P. Das, G. Dezoort, P. Elmer, B. Greenberg, N. Haubrich, S. Higginbotham, A. Kalogeropoulos, G. Kopp, S. Kwan, D. Lange, M. T. Lucchini, J. Luo, D. Marlow, K. Mei, I. Ojalvo, J. Olsen, C. Palmer, P. Piroué, D. Stickland, C. Tully, S. Malik, S. Norberg, V. E. Barnes, R. Chawla, S. Das, L. Gutay, M. Jones, A. W. Jung, G. Negro, N. Neumeister, C. C. Peng, S. Piperov, A. Purohit, H. Qiu, J. F. Schulte, M. Stojanovic, N. Trevisani, F. Wang, A. Wildridge, R. Xiao, W. Xie, T. Cheng, J. Dolen, N. Parashar, A. Baty, S. Dildick, K. M. Ecklund, S. Freed, F. J. M. Geurts, M. Kilpatrick, A. Kumar, W. Li, B. P. Padley, R. Redjimi, J. Roberts, J. Rorie, W. Shi, A. G. Stahl Leiton, A. Bodek, P. de Barbaro, R. Demina, J. L. Dulemba, C. Fallon, T. Ferbel, M. Galanti, A. Garcia-Bellido, O. Hindrichs, A. Khukhunaishvili, E. Ranken, R. Taus, B. Chiarito, J. P. Chou, A. Gandrakota, Y. Gershtein, E. Halkiadakis, A. Hart, M. Heindl, E. Hughes, S. Kaplan, O. Karacheban, I. Laflotte, A. Lath, R. Montalvo, K. Nash, M. Osherson, S. Salur, S. Schnetzer, S. Somalwar, R. Stone, S. A. Thayil, S. Thomas, H. Wang, H. Acharya, A. G. Delannoy, S. Spanier, O. Bouhali, M. Dalchenko, A. Delgado, R. Eusebi, J. Gilmore, T. Huang, T. Kamon, H. Kim, S. Luo, S. Malhotra, R. Mueller, D. Overton, L. Perniè, D. Rathjens, A. Safonov, N. Akchurin, J. Damgov, V. Hegde, S. Kunori, K. Lamichhane, S. W. Lee, T. Mengke, S. Muthumuni, T. Peltola, S. Undleeb, I. Volobouev, Z. Wang, A. Whitbeck, E. Appelt, S. Greene, A. Gurrola, R. Janjam, W. Johns, C. Maguire, A. Melo, H. Ni, K. Padeken, F. Romeo, P. Sheldon, S. Tuo, J. Velkovska, M. W. Arenton, B. Cox, G. Cummings, J. Hakala, R. Hirosky, M. Joyce, A. Ledovskoy, A. Li, C. Neu, B. Tannenwald, Y. Wang, E. Wolfe, F. Xia, P. E. Karchin, N. Poudyal, P. Thapa, K. Black, T. Bose, J. Buchanan, C. Caillol, S. Dasu, I. De Bruyn, P. Everaerts, C. Galloni, H. He, M. Herndon, A. Hervé, U. Hussain, A. Lanaro, A. Loeliger, R. Loveless, J. Madhusudanan Sreekala, A. Mallampalli, D. Pinna, A. Savin, V. Shang, V. Sharma, W. H. Smith, D. Teague, S. Trembath-reichert, W. Vetens, CMS Collaboration

**Affiliations:** 1 Yerevan Physics Institute, Yerevan, Armenia; 2 Institut für Hochenergiephysik, Vienna, Austria; 3 Institute for Nuclear Problems, Minsk, Belarus; 4 Universiteit Antwerpen, Antwerpen, Belgium; 5 Vrije Universiteit Brussel, Brussel, Belgium; 6 Université Libre de Bruxelles, Bruxelles, Belgium; 7 Ghent University, Ghent, Belgium; 8 Université Catholique de Louvain, Louvain-la-Neuve, Belgium; 9 Centro Brasileiro de Pesquisas Fisicas, Rio de Janeiro, Brazil; 10 Universidade do Estado do Rio de Janeiro, Rio de Janeiro, Brazil; 11 Universidade Estadual Paulista (a), Universidade Federal do ABC (b), São Paulo, Brazil; 12 Institute for Nuclear Research and Nuclear Energy, Bulgarian Academy of Sciences, Sofia, Bulgaria; 13 University of Sofia, Sofia, Bulgaria; 14 Beihang University, Beijing, China; 15 Department of Physics, Tsinghua University, Beijing, China; 16 Institute of High Energy Physics, Beijing, China; 17 State Key Laboratory of Nuclear Physics and Technology, Peking University, Beijing, China; 18 Sun Yat-Sen University, Guangzhou, China; 19 Institute of Modern Physics and Key Laboratory of Nuclear Physics and Ion-beam Application (MOE), Fudan University, Shanghai, China; 20 Zhejiang University, Hangzhou, Zhejiang China; 21 Universidad de Los Andes, Bogota, Colombia; 22 Universidad de Antioquia, Medellin, Colombia; 23 Faculty of Electrical Engineering, Mechanical Engineering and Naval Architecture, University of Split, Split, Croatia; 24 Faculty of Science, University of Split, Split, Croatia; 25 Institute Rudjer Boskovic, Zagreb, Croatia; 26 University of Cyprus, Nicosia, Cyprus; 27 Charles University, Prague, Czech Republic; 28 Escuela Politecnica Nacional, Quito, Ecuador; 29 Universidad San Francisco de Quito, Quito, Ecuador; 30 Academy of Scientific Research and Technology of the Arab Republic of Egypt, Egyptian Network of High Energy Physics, Cairo, Egypt; 31 Center for High Energy Physics (CHEP-FU), Fayoum University, El-Fayoum, Egypt; 32 National Institute of Chemical Physics and Biophysics, Tallinn, Estonia; 33 Department of Physics, University of Helsinki, Helsinki, Finland; 34 Helsinki Institute of Physics, Helsinki, Finland; 35 Lappeenranta University of Technology, Lappeenranta, Finland; 36 IRFU, CEA, Université Paris-Saclay, Gif-sur-Yvette, France; 37 Laboratoire Leprince-Ringuet, CNRS/IN2P3, Ecole Polytechnique, Institut Polytechnique de Paris, Palaiseau, France; 38 Université de Strasbourg, CNRS, IPHC UMR 7178, Strasbourg, France; 39 Institut de Physique des 2 Infinis de Lyon (IP2I ), Villeurbanne, France; 40 Georgian Technical University, Tbilisi, Georgia; 41 I. Physikalisches Institut, RWTH Aachen University, Aachen, Germany; 42 III. Physikalisches Institut A, RWTH Aachen University, Aachen, Germany; 43 III. Physikalisches Institut B, RWTH Aachen University, Aachen, Germany; 44 Deutsches Elektronen-Synchrotron, Hamburg, Germany; 45 University of Hamburg, Hamburg, Germany; 46 Karlsruher Institut fuer Technologie, Karlsruhe, Germany; 47 Institute of Nuclear and Particle Physics (INPP), NCSR Demokritos, Aghia Paraskevi, Greece; 48 National and Kapodistrian University of Athens, Athens, Greece; 49 National Technical University of Athens, Athens, Greece; 50 University of Ioánnina, Ioannina, Greece; 51 MTA-ELTE Lendület CMS Particle and Nuclear Physics Group, Eötvös Loránd University, Budapest, Hungary; 52 Wigner Research Centre for Physics, Budapest, Hungary; 53 Institute of Nuclear Research ATOMKI, Debrecen, Hungary; 54 Institute of Physics, University of Debrecen, Debrecen, Hungary; 55 Karoly Robert Campus, MATE Institute of Technology, Gyongyos, Hungary; 56 Indian Institute of Science (IISc), Bangalore, India; 57 National Institute of Science Education and Research, HBNI, Bhubaneswar, India; 58 Panjab University, Chandigarh, India; 59 University of Delhi, Delhi, India; 60 Saha Institute of Nuclear Physics, HBNI, Kolkata, India; 61 Indian Institute of Technology Madras, Madras, India; 62 Bhabha Atomic Research Centre, Mumbai, India; 63 Tata Institute of Fundamental Research-A, Mumbai, India; 64 Tata Institute of Fundamental Research-B, Mumbai, India; 65 Indian Institute of Science Education and Research (IISER), Pune, India; 66 Isfahan University of Technology, Isfahan, Iran; 67 Institute for Research in Fundamental Sciences (IPM), Tehran, Iran; 68 University College Dublin, Dublin, Ireland; 69 INFN Sezione di Bari, Bari, Italy, Università di Bari, Bari, Italy, Politecnico di Bari, Bari, Italy; 70 INFN Sezione di Bologna, Bologna, Italy, Università di Bologna, Bologna, Italy; 71 INFN Sezione di Catania, Catania, Italy, Università di Catania, Catania, Italy; 72 INFN Sezione di Firenze, Firenze, Italy, Università di Firenze, Firenze, Italy; 73 INFN Laboratori Nazionali di Frascati, Frascati, Italy; 74 INFN Sezione di Genova, Genova, Italy, Università di Genova, Genoa, Italy; 75 INFN Sezione di Milano-Bicocca, Milano, Italy, Università di Milano-Bicocca, Milan, Italy; 76 INFN Sezione di Napoli, Napoli, Italy, Università di Napoli ’Federico II’, Napoli, Italy, Università della Basilicata, Potenza, Italy, Università G. Marconi, Rome, Italy; 77 INFN Sezione di Padova, Padova, Italy, Università di Padova, Padova, Italy, Università di Trento, Trento, Italy; 78 INFN Sezione di Pavia, Pavia, Italy, Università di Pavia, Pavia, Italy; 79 INFN Sezione di Perugia, Perugia, Italy, Università di Perugia, Perugia, Italy; 80 INFN Sezione di Pisa, Pisa, Italy, Università di Pisa, Pisa, Italy, Scuola Normale Superiore di Pisa, Pisa, Italy, Università di Siena, Siena, Italy; 81 INFN Sezione di Roma, Rome, Italy, Sapienza Università di Roma, Rome, Italy; 82 INFN Sezione di Torino, Torino, Italy, Università di Torino, Torino, Italy, Università del Piemonte Orientale, Novara, Italy; 83 INFN Sezione di Trieste, Trieste, Italy, Università di Trieste, Trieste, Italy; 84 Kyungpook National University, Daegu, Korea; 85 Chonnam National University, Institute for Universe and Elementary Particles, Kwangju, Korea; 86 Hanyang University, Seoul, Korea; 87 Korea University, Seoul, Korea; 88 Department of Physics, Kyung Hee University, Seoul, Republic of Korea; 89 Sejong University, Seoul, Korea; 90 Seoul National University, Seoul, Korea; 91 University of Seoul, Seoul, Korea; 92 Department of Physics, Yonsei University, Seoul, Korea; 93 Sungkyunkwan University, Suwon, Korea; 94 College of Engineering and Technology, American University of the Middle East (AUM), Egaila, Dasman, Kuwait; 95 Riga Technical University, Riga, Latvia; 96 Vilnius University, Vilnius, Lithuania; 97 National Centre for Particle Physics, Universiti Malaya, Kuala Lumpur, Malaysia; 98 Universidad de Sonora (UNISON), Hermosillo, Mexico; 99 Centro de Investigacion y de Estudios Avanzados del IPN, Mexico City, Mexico; 100 Universidad Iberoamericana, Mexico City, Mexico; 101 Benemerita Universidad Autonoma de Puebla, Puebla, Mexico; 102 Universidad Autónoma de San Luis Potosí, San Luis Potosí, Mexico; 103 University of Montenegro, Podgorica, Montenegro; 104 University of Auckland, Auckland, New Zealand; 105 University of Canterbury, Christchurch, New Zealand; 106 National Centre for Physics, Quaid-I-Azam University, Islamabad, Pakistan; 107 AGH University of Science and Technology Faculty of Computer Science, Electronics and Telecommunications, Kraków, Poland; 108 National Centre for Nuclear Research, Swierk, Poland; 109 Institute of Experimental Physics, Faculty of Physics, University of Warsaw, Warsaw, Poland; 110 Laboratório de Instrumentação e Física Experimental de Partículas, Lisbon, Portugal; 111 Joint Institute for Nuclear Research, Dubna, Russia; 112 Petersburg Nuclear Physics Institute, Gatchina (St. Petersburg), Russia; 113 Institute for Nuclear Research, Moscow, Russia; 114 Institute for Theoretical and Experimental Physics named by A.I. Alikhanov of NRC ‘Kurchatov Institute’, Moscow, Russia; 115 Moscow Institute of Physics and Technology, Moscow, Russia; 116 National Research Nuclear University ‘Moscow Engineering Physics Institute’ (MEPhI), Moscow, Russia; 117 P.N. Lebedev Physical Institute, Moscow, Russia; 118 Skobeltsyn Institute of Nuclear Physics, Lomonosov Moscow State University, Moscow, Russia; 119 Novosibirsk State University (NSU), Novosibirsk, Russia; 120 Institute for High Energy Physics of National Research Centre ‘Kurchatov Institute’, Protvino, Russia; 121 National Research Tomsk Polytechnic University, Tomsk, Russia; 122 Tomsk State University, Tomsk, Russia; 123 University of Belgrade: Faculty of Physics and VINCA Institute of Nuclear Sciences, Belgrade, Serbia; 124 Centro de Investigaciones Energéticas Medioambientales y Tecnológicas (CIEMAT), Madrid, Spain; 125 Universidad Autónoma de Madrid, Madrid, Spain; 126 Instituto Universitario de Ciencias y Tecnologías Espaciales de Asturias (ICTEA), Universidad de Oviedo, Oviedo, Spain; 127 Instituto de Física de Cantabria (IFCA), CSIC-Universidad de Cantabria, Santander, Spain; 128 University of Colombo, Colombo, Sri Lanka; 129 Department of Physics, University of Ruhuna, Matara, Sri Lanka; 130 CERN, European Organization for Nuclear Research, Geneva, Switzerland; 131 Paul Scherrer Institut, Villigen, Switzerland; 132 ETH Zurich-Institute for Particle Physics and Astrophysics (IPA), Zurich, Switzerland; 133 Universität Zürich, Zurich, Switzerland; 134 National Central University, Chung-Li, Taiwan; 135 National Taiwan University (NTU), Taipei, Taiwan; 136 Department of Physics, Faculty of Science, Chulalongkorn University, Bangkok, Thailand; 137 Physics Department, Science and Art Faculty, Çukurova University, Adana, Turkey; 138 Physics Department, Middle East Technical University, Ankara, Turkey; 139 Bogazici University, Istanbul, Turkey; 140 Istanbul Technical University, Istanbul, Turkey; 141 Istanbul University, Istanbul, Turkey; 142 Institute for Scintillation Materials of National Academy of Science of Ukraine, Kharkov, Ukraine; 143 National Scientific Center, Kharkov Institute of Physics and Technology, Kharkov, Ukraine; 144 University of Bristol, Bristol, UK; 145 Rutherford Appleton Laboratory, Didcot, UK; 146 Imperial College, London, UK; 147 Brunel University, Uxbridge, UK; 148 Baylor University, Waco, TX USA; 149 Catholic University of America, Washington, DC USA; 150 The University of Alabama, Tuscaloosa, AL USA; 151 Boston University, Boston, MA USA; 152 Brown University, Providence, Rhode Island USA; 153 University of California, Davis, Davis, CA USA; 154 University of California, Los Angeles, CA USA; 155 University of California, Riverside, Riverside, CA USA; 156 University of California, San Diego, La Jolla, CA USA; 157 Department of Physics, University of California, Santa Barbara, Santa Barbara, CA USA; 158 California Institute of Technology, Pasadena, CA USA; 159 Carnegie Mellon University, Pittsburgh, PA USA; 160 University of Colorado Boulder, Boulder, CO USA; 161 Cornell University, Ithaca, NY USA; 162 Fermi National Accelerator Laboratory, Batavia, IL USA; 163 University of Florida, Gainesville, FL USA; 164 Florida State University, Tallahassee, FL USA; 165 Florida Institute of Technology, Melbourne, FL USA; 166 University of Illinois at Chicago (UIC), Chicago, IL USA; 167 The University of Iowa, Iowa City, IA USA; 168 Johns Hopkins University, Baltimore, MD USA; 169 The University of Kansas, Lawrence, KS USA; 170 Kansas State University, Manhattan, KS USA; 171 Lawrence Livermore National Laboratory, Livermore, CA USA; 172 University of Maryland, College Park, MD USA; 173 Massachusetts Institute of Technology, Cambridge, MA USA; 174 University of Minnesota, Minneapolis, MN USA; 175 University of Mississippi, Oxford, MS USA; 176 University of Nebraska-Lincoln, Lincoln, NE USA; 177 State University of New York at Buffalo, Buffalo, NY USA; 178 Northeastern University, Boston, MA USA; 179 Northwestern University, Evanston, IL USA; 180 University of Notre Dame, Notre Dame, IN USA; 181 The Ohio State University, Columbus, OH USA; 182 Princeton University, Princeton, NJ USA; 183 University of Puerto Rico, Mayaguez, PR USA; 184 Purdue University, West Lafayette, IN USA; 185 Purdue University Northwest, Hammond, IN USA; 186 Rice University, Houston, TX USA; 187 University of Rochester, Rochester, NY USA; 188 Rutgers, The State University of New Jersey, Piscataway, NJ USA; 189 University of Tennessee, Knoxville, TN USA; 190 Texas A&M University, College Station, TX USA; 191 Texas Tech University, Lubbock, TX USA; 192 Vanderbilt University, Nashville, TN USA; 193 University of Virginia, Charlottesville, VA USA; 194 Wayne State University, Detroit, MI USA; 195 University of Wisconsin-Madison, Madison, WI USA; 196 TU Wien, Wien, Austria; 197 Institute of Basic and Applied Sciences, Faculty of Engineering, Arab Academy for Science Technology and Maritime Transport, Alexandria, Egypt; 198 Université Libre de Bruxelles, Bruxelles, Belgium; 199 IRFU, CEA, Université Paris-Saclay, Gif-sur-Yvette, France; 200 Universidade Estadual de Campinas, Campinas, Brazil; 201 Federal University of Rio Grande do Sul, Porto Alegre, Brazil; 202 UFMS, Nova Andradina, Brazil; 203 Universidade Federal de Pelotas, Pelotas, Brazil; 204 Department of Physics, Nanjing Normal University, Nanjing, China; 205 University of Chinese Academy of Sciences, Beijing, China; 206 Institute for Theoretical and Experimental Physics named by A.I. Alikhanov of NRC ‘Kurchatov Institute’, Moscow, Russia; 207 Joint Institute for Nuclear Research, Dubna, Russia; 208 Cairo University, Cairo, Egypt; 209 Suez University, Suez, Egypt; 210 Now at British University in Egypt, Cairo, Egypt; 211 Zewail City of Science and Technology, Zewail, Egypt; 212 Purdue University, West Lafayette, IN USA; 213 Université de Haute Alsace, Mulhouse, France; 214 Erzincan Binali Yildirim University, Erzincan, Turkey; 215 CERN, European Organization for Nuclear Research, Geneva, Switzerland; 216 III. Physikalisches Institut A, RWTH Aachen University, Aachen, Germany; 217 University of Hamburg, Hamburg, Germany; 218 Isfahan University of Technology, Isfahan, Iran; 219 Brandenburg University of Technology, Cottbus, Germany; 220 Skobeltsyn Institute of Nuclear Physics, Lomonosov Moscow State University, Moscow, Russia; 221 Institute of Physics, University of Debrecen, Debrecen, Hungary; 222 Physics Department, Faculty of Science, Assiut University, Assiut, Egypt; 223 MTA-ELTE Lendület CMS Particle and Nuclear Physics Group, Eötvös Loránd University, Budapest, Hungary; 224 Institute of Nuclear Research ATOMKI, Debrecen, Hungary; 225 IIT Bhubaneswar, Bhubaneswar, India; 226 Institute of Physics, Bhubaneswar, India; 227 G.H.G. Khalsa College, Punjab, India; 228 Shoolini University, Solan, India; 229 University of Hyderabad, Hyderabad, India; 230 University of Visva-Bharati, Santiniketan, India; 231 Indian Institute of Technology (IIT), Mumbai, India; 232 Deutsches Elektronen-Synchrotron, Hamburg, Germany; 233 Sharif University of Technology, Tehran, Iran; 234 Department of Physics, University of Science and Technology of Mazandaran, Behshahr, Iran; 235 Now at INFN Sezione di Bari, Bari, Italy, Università di Bari, Bari, Italy, Politecnico di Bari, Bari, Italy; 236 Italian National Agency for New Technologies, Energy and Sustainable Economic Development, Bologna, Italy; 237 Centro Siciliano di Fisica Nucleare e di Struttura Della Materia, Catania, Italy; 238 Università di Napoli ’Federico II’, Naples, Italy; 239 Riga Technical University, Riga, Latvia; 240 Consejo Nacional de Ciencia y Tecnología, Mexico City, Mexico; 241 Institute for Nuclear Research, Moscow, Russia; 242 Now at National Research Nuclear University ‘Moscow Engineering Physics Institute’ (MEPhI), Moscow, Russia; 243 Institute of Nuclear Physics of the Uzbekistan Academy of Sciences, Tashkent, Uzbekistan; 244 St. Petersburg State Polytechnical University, St. Petersburg, Russia; 245 University of Florida, Gainesville, Florida, USA; 246 Imperial College, London, UK; 247 Moscow Institute of Physics and Technology, Moscow, Russia; 248 P.N. Lebedev Physical Institute, Moscow, Russia; 249 California Institute of Technology, Pasadena, California, USA; 250 Budker Institute of Nuclear Physics, Novosibirsk, Russia; 251 Faculty of Physics, University of Belgrade, Belgrade, Serbia; 252 Trincomalee Campus, Eastern University, Nilaveli, Sri Lanka; 253 INFN Sezione di Pavia, Pavia, Italy, Università di Pavia, Pavia, Italy; 254 National and Kapodistrian University of Athens, Athens, Greece; 255 Universität Zürich, Zurich, Switzerland; 256 Ecole Polytechnique Fédérale Lausanne, Lausanne, Switzerland; 257 Stefan Meyer Institute for Subatomic Physics, Vienna, Austria; 258 Laboratoire d’Annecy-le-Vieux de Physique des Particules, IN2P3-CNRS, Annecy-le-Vieux, France; 259 Şırnak University, Sirnak, Turkey; 260 Department of Physics, Tsinghua University, Beijing, China; 261 Near East University, Research Center of Experimental Health Science, Nicosia, Turkey; 262 Beykent University, Istanbul, Turkey; 263 Istanbul Aydin University, Application and Research Center for Advanced Studies, Istanbul, Turkey; 264 Mersin University, Mersin, Turkey; 265 Piri Reis University, Istanbul, Turkey; 266 Adiyaman University, Adiyaman, Turkey; 267 Ozyegin University, Istanbul, Turkey; 268 Izmir Institute of Technology, Izmir, Turkey; 269 Necmettin Erbakan University, Konya, Turkey; 270 Bozok Universitetesi Rektörlügü, Yozgat, Turkey; 271 Marmara University, Istanbul, Turkey; 272 Milli Savunma University, Istanbul, Turkey; 273 Kafkas University, Kars, Turkey; 274 Istanbul Bilgi University, Istanbul, Turkey; 275 Hacettepe University, Ankara, Turkey; 276 Vrije Universiteit Brussel, Brussel, Belgium; 277 School of Physics and Astronomy, University of Southampton, Southampton, UK; 278 IPPP Durham University, Durham, UK; 279 Monash University, Faculty of Science, Clayton, Australia; 280 Bethel University, St. Paul, Minneapolis, USA; 281 Karamanoğlu Mehmetbey University, Karaman, Turkey; 282 Ain Shams University, Cairo, Egypt; 283 Bingol University, Bingol, Turkey; 284 Georgian Technical University, Tbilisi, Georgia; 285 Sinop University, Sinop, Turkey; 286 Mimar Sinan University, Istanbul, Turkey; 287 Texas A&M University at Qatar, Doha, Qatar; 288 Kyungpook National University, Daegu, Korea; 289 CERN, 1211 Geneva 23, Switzerland

## Abstract

A search for low-mass dilepton resonances in Higgs boson decays is conducted in the four-lepton final state. The decay is assumed to proceed via a pair of beyond the standard model particles, or one such particle and a ${\mathrm{Z}}$ boson. The search uses proton–proton collision data collected with the CMS detector at the CERN LHC, corresponding to an integrated luminosity of 137$\,\text {fb}^{-1}$, at a center-of-mass energy $\sqrt{s} = 13\,\text {TeV}$. No significant deviation from the standard model expectation is observed. Upper limits at 95% confidence level are set on model-independent Higgs boson decay branching fractions. Additionally, limits on dark photon and axion-like particle production, based on two specific models, are reported.

## Introduction

Following the discovery of the Higgs boson (${\mathrm{H}}$) by the ATLAS and CMS Collaborations [1–3] at the CERN LHC, a thorough program of precise measurements [4–6] has been carried out to uncover possible deviations from the standard model (SM) or to decipher the nature of the Higgs sector. In particular, various exotic decays of the Higgs boson have been considered, in which small deviations in the Higgs boson decay width or discovery of exotic decay modes could constitute evidence of beyond the SM (BSM) physics.

This paper describes a search for exotic decays of the Higgs boson ${{{\mathrm{H}}} \rightarrow {{\mathrm{Z}}} {{\mathrm{X}}}}$ or ${{{\mathrm{H}}} \rightarrow {{\mathrm{X}}} {{\mathrm{X}}}}$ in the four-lepton (electrons or muons) final state, using a sample of proton–proton collision data at a center-of-mass energy of 13$\,\text {TeV}$ recorded by the CMS experiment in 2016–2018. The analyzed data sample corresponds to an integrated luminosity of $137{\,\text {fb}^{-1}}$. Here ${\mathrm{X}}$ represents a possible BSM particle that could decay into a pair of opposite-sign, same-flavor (OSSF) leptons. In this paper, we consider two specific BSM models. In both models, leptonic decays of ${\mathrm{X}}$ and ${\mathrm{Z}}$ to either two muons or electrons give rise to the 4$\ell$ (where $4\ell$ may denote $4{{\mathrm{\upmu }}}$, $2{{\mathrm{e}}} 2{{\mathrm{\upmu }}}$, or $4{{\mathrm{e}}}$) final states. Assuming narrow-width approximation decays of ${\mathrm{X}}$, only the mass range $m_{{\mathrm{X}}} < m_{{\mathrm{H}}}- m_{{\mathrm{Z}}} \approx 35\,\text {GeV}$ ($m_{{\mathrm{X}}} < m_{{\mathrm{H}}}/2 \approx 62.5\,\text {GeV}$) is kinematically possible for ${{\mathrm{H}}} \rightarrow {{\mathrm{Z}}} {{\mathrm{X}}}$ (${{\mathrm{H}}} \rightarrow {{\mathrm{X}}} {{\mathrm{X}}}$), where $m_{{{\mathrm{H}}}}$ and $m_{{{\mathrm{Z}}}}$ are the Higgs boson mass and Z boson mass, respectively. The decay channel ${{\mathrm{p}}} {{\mathrm{p}}} \rightarrow {{\mathrm{H}}} \rightarrow 4\ell$ has a large signal-to-background ratio. This channel allows a complete reconstruction of the kinematics of the Higgs boson based on final-state decay particles. In this analysis, a mass range of $4.0< m_{{{\mathrm{X}}}} < 35.0\,\text {GeV}$ (62.5$\,\text {GeV}$) is considered.

**Fig. 1:**
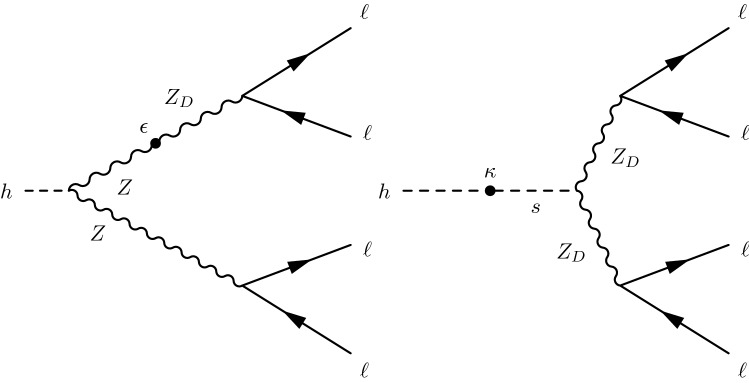
Feynman diagrams for Higgs boson decay via the kinetic-mixing (left) or Higgs-mixing mechanism (right) [7]. The symbol ${{\mathrm{h}}}$ represents the Higgs boson, and *s* represents the dark Higgs boson. The symbol $\varepsilon$ represents the kinetic-mixing parameter while $\kappa$ represents the Higgs-mixing parameter

The first model considered, hereby referred to as the “hidden Abelian Higgs model” (HAHM), concerns theories with a hidden “dark” sector [7–11], with the X particle identified as the dark photon (${{\mathrm{Z}}} _{{\mathrm {D}}}$), which mediates a dark $U(1)_{D}$ gauge symmetry, which is spontaneously broken by a dark Higgs mechanism. Interactions of the dark sector with SM particles can occur through a hypercharge portal via the kinetic-mixing parameter $\varepsilon$, or through a Higgs portal via the Higgs-mixing parameter $\kappa$, as shown in Fig. 1. Details of this theory and subsequent phenomenological implications can be found in Ref. [7]. Several searches for ${{\mathrm{Z}}} _{{\mathrm {D}}}$ were previously performed by collider experiments, for example ATLAS [12, 13] and LHCb [14]. Other experiments, such as beam dump experiments, fixed target experiments, helioscopes, and cold dark matter direct detection experiments, provide complementary sensitivities to ${{\mathrm{Z}}} _{{\mathrm {D}}}$. A summary of the experimental coverage of the HAHM model can be found in Refs. [15, 16].

The second model involves axion-like particles (ALPs), with ${\mathrm{X}}$ being a pseudoscalar gauge singlet ${\mathrm{a}}$. Axions were originally proposed to address the strong CP problem [17]. Recently, ALPs were proposed to explain the observed anomaly in the magnetic moment of the muon [18]. Theoretical overviews of the ALP models can be found in Refs. [19, 20]. The models are formulated as an effective field theory of ALPs coupled to various SM particles. In particular, the theory allows the coupling between the Higgs boson, Z boson, and the ALP field, or the Higgs boson and the ALP field. These couplings are represented by the Wilson coefficients $C_{{{\mathrm{Z}}} {{\mathrm{H}}}}/\varLambda$ and $C_{{{\mathrm{a}}} {{\mathrm{H}}}}/\varLambda ^2$, respectively, where $\varLambda$ is the decoupling energy scale in the effective field theory, or the mass scale of new physics. The former (latter) coefficient gives rise to the exotic decay of ${{\mathrm{H}}} \rightarrow {{\mathrm{Z}}} {{\mathrm{a}}}$ (${{\mathrm{a}}} {{\mathrm{a}}}$). Various experimental searches for ${{\mathrm{H}}} \rightarrow {{\mathrm{a}}} {{\mathrm{a}}}$ have been performed [21–26]. Recently a direct search for ${{\mathrm{H}}} \rightarrow {{\mathrm{Z}}} {{\mathrm{a}}}$ has been performed targeting a signature with a light and hadronically decaying resonance ${{\mathrm{a}}}$ with ${m_{{{\mathrm{a}}}} < 4\,\text {GeV}}$ [27]. The present search provides complementary coverage of the phase space of the ALP model with mass greater than 4$\,\text {GeV}$.

This paper is organized as follows. Section 2 describes the CMS detector and event reconstruction algorithms. Section 3 outlines the collision data used and various software packages used to generate the samples of simulated events. Section 4 summarizes the selection criteria and the categorization of signal events, and Sect. 5 describes the reducible background estimation method. Section 6 describes the various sources of systematic uncertainties in the search. Finally, results and interpretations are detailed in Sect. 7, and a summary is given in Sect. 8. Tabulated results are provided in HEPData [28].

## The CMS detector and event reconstruction

The central feature of the CMS apparatus is a superconducting solenoid of 6$\,\text {m}$ internal diameter, providing a magnetic field of 3.8$\,\text {T}$. Within the solenoid volume are a silicon pixel and strip tracker, a lead tungstate crystal electromagnetic calorimeter (ECAL), and a brass and scintillator hadron calorimeter (HCAL), each composed of a barrel and two endcap sections. Forward calorimeters extend the pseudorapidity ($\eta$) coverage provided by the barrel and endcap detectors. Muons are detected in gas-ionization chambers embedded in the steel flux-return yoke outside the solenoid. A more detailed description of the CMS detector, together with a definition of the coordinate system used and the relevant kinematic variables, can be found in Ref. [29].

Events of interest are selected using a two-tiered trigger system [30]. The first level, composed of custom hardware processors, uses information from the calorimeters and muon detectors to select events at a rate of around 100$\,\text {kHz}$ within a fixed time interval of about 4$\,\upmu \text {s}$. The second level, known as the high-level trigger, consists of a farm of processors running a version of the full event reconstruction software optimized for fast processing, and reduces the event rate to around 1$\,\text {kHz}$ before data storage.

The candidate vertex with the largest value of summed physics-object $p_{{\mathrm {T}}} ^2$ (where $p_{{\mathrm {T}}}$ is the transverse momentum) is taken to be the primary ${{\mathrm{p}}} {{\mathrm{p}}}$ interaction vertex. The physics objects are the jets, clustered using the jet finding algorithm [31, 32] with the tracks assigned to candidate vertices as inputs, and the associated missing transverse momentum, taken as the negative vector sum of the $p_{{\mathrm {T}}}$ of those jets.

The particle-flow (PF) algorithm [33] aims to reconstruct and identify each individual particle in an event (PF candidate), with an optimized combination of information from the various elements of the CMS detector. The energy of photons is obtained from the ECAL measurement. The energy of electrons is determined from a combination of the electron momentum at the primary interaction vertex as determined by the tracker, the energy of the corresponding ECAL cluster, and the energy sum of all bremsstrahlung photons spatially compatible with originating from the electron track. The energy of muons is obtained from the curvature of the corresponding track. The energy of charged hadrons is determined from a combination of their momentum measured in the tracker and the matching ECAL and HCAL energy deposits, corrected for the response function of the calorimeters to hadronic showers. Finally, the energy of neutral hadrons is obtained from the corresponding corrected ECAL and HCAL energies.

The missing transverse momentum vector ${\vec p}_{{\mathrm {T}}}^{\text {miss}}$ is computed as the negative vector sum of the transverse momenta of all the PF candidates in an event, and its magnitude is denoted as $p_{{\mathrm {T}}} ^\text {miss}$  [34]. The ${\vec p}_{{\mathrm {T}}}^{\text {miss}}$ is modified to account for corrections to the energy scale of the reconstructed jets in the event.

Muons in the four lepton final state are measured in the range ${|\eta | < 2.4}$, with detection planes made using three technologies: drift tubes, cathode strip chambers, and resistive plate chambers. The single-muon trigger efficiency exceeds 90% over the full $\eta$ range, and the efficiency to reconstruct and identify muons is greater than 96%. Matching muons to tracks measured in the silicon tracker results in a relative $p_{{\mathrm {T}}}$ resolution, for muons with $p_{{\mathrm {T}}}$ up to 100$\,\text {GeV}$, of 1% in the barrel and 3% in the endcaps [35].

Electrons in the four lepton final state with ${p_{{\mathrm {T}}} > 7\,\text {GeV}}$ and ${|\eta | < 2.5}$ are identified by a multivariate discriminant, which is constructed by observables related to the bremsstrahlung along the electron trajectory, ECAL energy measurements, electromagnetic showers, missing pixel detector hits, and the photon conversion vertex fit probability [36]. The electron momentum is estimated by combining the energy measurement in the ECAL with the momentum measurement in the tracker. The momentum resolution for electrons with ${p_{{\mathrm {T}}} \approx 45\,\text {GeV}}$ from ${{{\mathrm{Z}}} \rightarrow {{\mathrm{e}}} {{\mathrm{e}}}}$ decays ranges from 1.7 to 4.5%. It is generally better in the barrel region than in the endcaps, and also depends on the bremsstrahlung energy emitted by the electron as it traverses the material in front of the ECAL. The dielectron mass resolution for ${{{\mathrm{Z}}} \rightarrow {{\mathrm{e}}} {{\mathrm{e}}}}$ decays when both electrons are in the ECAL barrel (endcap) is 1.9% (2.9%).

This analysis focuses on promptly produced signal processes. To reduce the contributions from leptons arising from hadron decays within jets, a requirement is imposed on each lepton candidate using a variable defined as:

1 $$\begin{aligned} I^{\ell } = \frac{\sum p_{{\mathrm {T}}} ^{\text {charged}}+\max \Bigl [0,\sum p_{{\mathrm {T}}} ^{\text {neutral}}+\sum p_{{\mathrm {T}}} ^{{{\mathrm{\upgamma }}}}-p_{{\mathrm {T}}} ^{\text {PU}}\Bigr ]}{p_{{\mathrm {T}}} ^{\ell }} \end{aligned}$$

where the sums are over the PF candidates within a cone of radius ${R = \sqrt{\smash [b]{\varDelta \eta ^2+\varDelta \phi ^2}} < 0.3}$ (where $\phi$ is the azimuthal angle in radians), $p_{{\mathrm {T}}} ^i$ represents transverse momenta from each particle *i*, where *i* represents either charged hadrons, neutral hadrons, photons, or particles originating from overlapping proton–proton interactions (pileup) [37]. For muons, the isolation is required to be ${I^{{{\mathrm{\upmu }}}} < 0.35}$. For electrons, this variable is included in the multivariate discriminant for datasets in 2017 and 2018, while for the dataset in 2016, an isolation requirement ${I^{{{\mathrm{e}}}} < 0.35}$ is imposed on each electron candidate. In addition, the three-dimensional impact parameter of electrons and muons is required to be consistent with the primary collision vertex. The requirement implies a negligible acceptance to signal models with long-lived ${\mathrm{X}}$.

An algorithm is utilized to correct for effects arising from final-state radiation (FSR) from leptons. PF-reconstructed photons are considered as FSR candidates if they satisfy the requirement ${p_{{\mathrm {T}}} ^{{{\mathrm{\upgamma }}}} > 2\,\text {GeV}}$ and ${I^{{{\mathrm{\upgamma }}}} < 1.8}$, where $I^{{{\mathrm{\upgamma }}}}$ is calculated similarly to the lepton isolation variable. Then each FSR candidate is assigned to the closest lepton in the event. The candidates are further required to have ${\varDelta R({{\mathrm{\upgamma }}},\ell )/(p_{{\mathrm {T}}} ^{{{\mathrm{\upgamma }}}})^2 < 0.012\,\text {GeV} ^{-2}}$ and ${\varDelta R({{\mathrm{\upgamma }}},\ell ) < 0.5}$. These candidates are excluded from the calculation of the lepton isolation variables.

Lepton reconstruction and selection efficiencies are measured in data by a “tag-and-probe” technique with an inclusive sample of ${\mathrm{Z}}$ boson events [38]. The difference between the efficiencies in data and simulation are observed to be around 1–4%, depending on $p_{{\mathrm {T}}}$ and $\eta$ of the lepton considered. The differences are used to correct lepton efficiencies in simulation.

## Data and simulated samples

Leading order (LO) signal samples for the physics processes ${{\mathrm{p}}} {{\mathrm{p}}} \rightarrow {{\mathrm{H}}} \rightarrow {{\mathrm{Z}}} {{\mathrm{Z}}} _{{\mathrm {D}}} ({{\mathrm{Z}}} _{{\mathrm {D}}} {{\mathrm{Z}}} _{{\mathrm {D}}}) \rightarrow 4\ell$, where $\ell = ({{\mathrm{e}}},{{\mathrm{\upmu }}})$, are generated using the MadGraph 5_amc@nlo 2.2.2 (2.4.2) [39–41] generator for 2016 (2017 and 2018), with HAHM [7] at leading order. Cross sections for each ${{\mathrm{Z}}} _{{\mathrm {D}}}$ signal are calculated by multiplying the next-to-next-to-next-to-leading order (NNNLO) Higgs production cross section [42] by the branching fraction of ${{\mathrm{H}}} \rightarrow {{\mathrm{Z}}} {{\mathrm{Z}}} _{{\mathrm {D}}}$ and ${{\mathrm{H}}} \rightarrow {{\mathrm{Z}}} _{{\mathrm {D}}} {{\mathrm{Z}}} _{{\mathrm {D}}}$, respectively [7]. Final states with $\tau$ leptons are neglected as their contribution to the signal region yield is below $1\%$. Signal contributions from vector-boson fusion and associated production with a top quark pair or a vector boson are also omitted.

The SM Higgs boson simulation samples, which include gluon fusion, vector boson fusion, and associated production with a top quark pair or a vector boson, and the simulated ${{\mathrm{Z}}} {{\mathrm{Z}}}$ background from quark-antiquark annihilation are generated at next-to-leading order (NLO) in perturbative quantum chromodynamics with powheg  v2 [43–46]. The cross section for the dominant production mode, gluon fusion, is taken at NNNLO [42].

Decays of the Higgs boson to four leptons are simulated with JHUGen 7.0.2 [47, 48]. The non-resonant process of ${{\mathrm{g}}} {{\mathrm{g}}} \rightarrow {{\mathrm{Z}}} {{\mathrm{Z}}}$ process is simulated at LO with mcfm 7.0.1 [49]. NLO correction factors [50] are applied to the ${{\mathrm{g}}} {{\mathrm{g}}} \rightarrow {{\mathrm{Z}}} {{\mathrm{Z}}}$ process.

Minor backgrounds from ${{\mathrm{t}}} {{{\overline{\mathrm{{{\mathrm{t}}}}}}}} {{\mathrm{Z}}}$ and triboson production processes are also simulated at LO and NLO, respectively, with the MadGraph 5_amc@nlo 2.2.2 (2.4.2) [39–41] generator for 2016 (2017 and 2018).

The set of parton distribution functions (PDFs) used was NNPDF3.0 [51] (NNPDF3.1 [52]) for the 2016 (2017 and 2018) simulation. Parton showering and hadronization are simulated using the pythia  8.230 generator [53] with the CUETP8M1 (CP5) underlying event tune for the 2016 (2017 and 2018) simulation [54, 55]. The response of the CMS detector is modeled using the Geant4 program [56, 57]. Simulated events are reweighted according to a specified instantaneous luminosity and an average number of pileup events.

## Event selection

In the trigger system, events are required to have more than two leptons. The overall trigger efficiency is measured in data using a sample of $4\ell$ events from single-lepton triggers and agreements are observed with simulation within 5%, and is found to be larger than 99%.

A set of requirements is applied to maximize the sensitivity of the search for a potential signal in the ${{\mathrm{Z}}} {{\mathrm{X}}}$ and ${{\mathrm{X}}} {{\mathrm{X}}}$ event topologies. In both cases, at least four well-identified and isolated leptons from the primary vertex are required, possibly accompanied by an FSR photon. Each muon (electron) is required to have $p_{{\mathrm {T}}} > 5\,\text {GeV}$ (7$\,\text {GeV}$). All four leptons must be separated from each other by $\varDelta R(\ell _i,\ell _j) > 0.02$. The leading (subleading) lepton $p_{{\mathrm {T}}}$ is required to satisfy $p_{{\mathrm {T}}} > 20\,\text {GeV}$ (10$\,\text {GeV}$). The four-lepton invariant mass $m_{4\ell }$ is required to be within $118< m_{4\ell } < 130\,\text {GeV}$. To further suppress background contributions from hadron decays in jet fragmentation or from the decay of low-mass resonances, all opposite-charge leptons pairs, regardless of lepton flavor, are required to satisfy $m_{\ell ^+ \ell ^-} > 4\,\text {GeV}$.

For each event in the ${{\mathrm{Z}}} {{\mathrm{X}}}$ and ${{\mathrm{X}}} {{\mathrm{X}}}$ searches, dilepton pair candidates are formed by considering all OSSF leptons. The dilepton invariant mass $m_{\ell ^{+} \ell ^{-}}$ for each candidate is required to be within $4< m_{\ell ^{+}\ell ^{-}} < 120\,\text {GeV}$, however the mass window around the $\varUpsilon$ ${{\mathrm{b}}} {{{\overline{\mathrm{{{\mathrm{b}}}}}}}}$ bound states ($8.0< m_{\varUpsilon } < 11.5\,\text {GeV}$) is also excluded.

Two dilepton candidates are then paired to form a ${{\mathrm{Z}}} {{\mathrm{X}}}$ or ${{\mathrm{X}}} {{\mathrm{X}}}$ event candidate. For the ${{\mathrm{Z}}} {{\mathrm{X}}}$ search, ${{\mathrm{Z}}} _1$ is the OSSF dilepton pair with an invariant mass closest to the ${\mathrm{Z}}$ boson mass [58] (representing ${\mathrm{Z}}$ in ${{\mathrm{Z}}} {{\mathrm{X}}}$), and ${{\mathrm{Z}}} _2$ is the other pair (${\mathrm{X}}$). For the ${{\mathrm{X}}} {{\mathrm{X}}}$ search, ${{\mathrm{Z}}} _1$ is the OSSF dilepton pair with the larger invariant mass, and ${{\mathrm{Z}}} _2$ is the lower-mass pair. For the ${{\mathrm{Z}}} {{\mathrm{X}}}$ search, $m_{{{\mathrm{Z}}} _1}$ is required to be larger than 40$\,\text {GeV}$. For the ${{\mathrm{X}}} {{\mathrm{X}}}$ search, $m_{{{\mathrm{Z}}} _1}$ and $m_{{{\mathrm{Z}}} _2}$ must lie between 4 and 62.5$\,\text {GeV}$. For events with more than four selected leptons, the combination of four leptons with $m_{{{\mathrm{Z}}} _1}$ closest to the Z boson is used for the ${{\mathrm{Z}}} {{\mathrm{X}}}$ candidate, while the combination with the least value of $(m_{{{\mathrm{Z}}} _1}-m_{{{\mathrm{Z}}} _2})/(m_{{{\mathrm{Z}}} _1} +m_{{{\mathrm{Z}}} _2})$ is used to select ${{\mathrm{X}}} {{\mathrm{X}}}$ candidates with similar invariant masses.

Four final-state lepton categories can be defined as $4{{\mathrm{\upmu }}}$, $2{{\mathrm{\upmu }}} 2{{\mathrm{e}}}$, $4{{\mathrm{e}}}$, $2{{\mathrm{e}}} 2{{\mathrm{\upmu }}}$, where the order of lepton flavors corresponds to Z1 and Z2 flavors. For the $4{{\mathrm{\upmu }}}$ and $4{{\mathrm{e}}}$ final states, one alternative pairing of the four leptons is possible, labelled by ${{\mathrm{Z}}} _\text {a}$ and ${{\mathrm{Z}}} _\text {b}$. For the ${{\mathrm{Z}}} {{\mathrm{X}}}$ search, events with $m_{{{\mathrm{Z}}} _\text {b}} < 12\,\text {GeV}$ and $m_{{{\mathrm{Z}}} _\text {a}}$ closer to the ${\mathrm{Z}}$ boson mass than ${{\mathrm{Z}}} _1$ are discarded to suppress background contributions from on-shell Z and low-mass dilepton resonances. For the ${{\mathrm{X}}} {{\mathrm{X}}}$ search, the ${{\mathrm{X}}} {{\mathrm{X}}}$ candidate with the smallest value of $(m_{{{\mathrm{Z}}} _1}-m_{{{\mathrm{Z}}} _2})/(m_{{{\mathrm{Z}}} _1} +m_{{{\mathrm{Z}}} _2})$ is chosen.

## Background estimation

### Irreducible background estimation

Irreducible backgrounds for this search come from processes including a SM Higgs boson, as well as nonresonant production of ${{\mathrm{Z}}} {{\mathrm{Z}}}$ via quark-antiquark annihilation or gluon fusion, and rare backgrounds such as ${{\mathrm{t}}} {{{\overline{\mathrm{{{\mathrm{t}}}}}}}} +{{\mathrm{Z}}}$ and triboson production. These backgrounds are estimated from simulation. Details of the simulation used for each of the backgrounds are described in Sect. 3.

### Reducible background estimation

The reducible backgrounds in the $4\ell$ final state can arise from the leptonic decays of heavy-flavor hadrons, in-flight decays of light mesons within jets, charged hadrons misidentified as electrons when in proximity of a $\uppi ^{0}$, and photon conversions. These backgrounds primarily arise from the ${{\mathrm{Z}}} +\text {jets}$ process. Additional physics processes with kinematics similar to the signal include ${{\mathrm{t}}} {{{\overline{\mathrm{{{\mathrm{t}}}}}}}}$, ${{\mathrm{Z}}} {{\mathrm{\upgamma }}}$, and ${{\mathrm{W}}} {{\mathrm{Z}}}$.

Two dedicated control regions are used to estimate the contribution from these backgrounds. The first (second) control region consists of events with two (three) leptons passing the lepton identification and isolation requirements and two (one) leptons failing the requirements, and is denoted as the 2P2F (3P1F) region. Backgrounds with only two prompt leptons, such as ${{\mathrm{Z}}} +\text {jets}$ and ${{\mathrm{t}}} {{{\overline{\mathrm{{{\mathrm{t}}}}}}}}$, are estimated by the 2P2F region, while backgrounds with three prompt leptons, such as ${{\mathrm{W}}} {{\mathrm{Z}}}$ and ${{\mathrm{Z}}} {{\mathrm{\upgamma }}}$ with the photon converting to an electron pair, are estimated by the 3P1F region. Other than the lepton requirements, the 3P1F and 2P2F regions follow the same event selection and alternative pairing algorithms as in the signal region to closely mimic its kinematics.

The lepton misidentification rates $f_{{{\mathrm{\upmu }}}}$ and $f_{{{\mathrm{e}}}}$ are measured as a function of lepton $p_{{\mathrm {T}}}$ and $\eta$ with a sample which includes a ${\mathrm{Z}}$ candidate, formed by a pair of leptons passing the selection requirement of the analysis, and an additional lepton passing a relaxed requirement. These rates are measured separately in the data samples from 2016, 2017, and 2018. In addition, the mass of the ${\mathrm{Z}}$ candidate is required to satisfy the condition $|m_{{{\mathrm{Z}}} _1}-m_{{{\mathrm{Z}}}} | < 7\,\text {GeV}$ to reduce contributions from ${{\mathrm{W}}} {{\mathrm{Z}}}$ and ${{\mathrm{t}}} {{{\overline{\mathrm{{{\mathrm{t}}}}}}}}$ processes, and $p_{{\mathrm {T}}} ^\text {miss}$ is required to be less than 25$\,\text {GeV}$.

To estimate the background contribution in the signal region, events in the 3P1F and 2P2F control regions are reweighted by lepton misidentification probabilities. Each event *i* in the 3P1F region is weighted by a factor $f^{i}_{4}/(1-f^{i}_{4})$, where $f^{i}_{4}$ corresponds to the lepton misidentification rate of the failed lepton in the event. Physics processes in the 2P2F control region can contribute to the 3P1F region and are estimated by reweighting 2P2F events with $f^{i}_{3}/(1-f^{i}_{3})+f^{i}_{4}/(1-f^{i}_{4})$, where $f^{i}_{3}$ and $f^{i}_{4}$ correspond to the lepton misidentification rates of the two failed leptons in the event. A minor contribution from ${{\mathrm{Z}}} {{\mathrm{Z}}}$ events to the 3P1F control region is estimated from simulation and subtracted. The expected yield for the signal region can then be estimated as:

2 $$\begin{aligned} N^{\text {reducible}}_{\mathrm {SR}}= & {} \left( 1-\frac{N^{{{\mathrm{Z}}} {{\mathrm{Z}}}}_{3P1F}}{N_{3P1F}} \right) \nonumber \\&\times \sum _{i}^{N_{3P1F}} \frac{f^{i}_{4}}{1-f^{i}_{4}} - \sum _{i}^{N_{2P2F}} \frac{f^{i}_{3}}{1-f^{i}_{3}} \frac{f^{i}_{4}}{1-f^{i}_{4}} \end{aligned}$$

where each sum is over all 3P1F and 2P2F events, respectively.

Furthermore, dedicated validation regions, which include adjacent $m_{4\ell }$ regions to the signal region ($70< m_{4\ell } < 118~\,\text {GeV}$, $130< m_{4\ell } < 200~\,\text {GeV}$), are defined to inspect the level of agreement between data and predictions.

## Systematic uncertainties

Experimental sources of the systematic uncertainties applicable to all final states include the integrated luminosity uncertainty and the lepton identification and reconstruction efficiency uncertainty. The integrated luminosities of the 2016, 2017, and 2018 data-taking periods are individually known with uncertainties in the 1.2–2.5% range [59–61], while the total Run 2 (2016–2018) integrated luminosity has an uncertainty of 1.6% [62], the improvement in precision reflecting the (uncorrelated) time evolution of some systematic effects. Lepton efficiency uncertainties are estimated in bins of lepton $p_{{\mathrm {T}}}$ and $\eta$ using the tag-and-probe method, as described in Sect. 2. These uncertainties on each lepton candidate lead to variations from 2.5 to 16.1% on event yields, dependent on final-state lepton categories. In addition, the systematic uncertainties in the lepton energy scale are determined by fitting the ${{\mathrm{Z}}} \rightarrow \ell \ell$ mass distribution in bins of lepton $p_{{\mathrm {T}}}$ and $\eta$ with a Breit–Wigner parameterization convolved with a double-sided Crystal Ball function [63]. Systematic uncertainties in the estimation of the reducible background are derived from the level of agreement between data and predictions in the validation regions in each lepton category (23–48% depending on data taking period), arising from different background compositions between signal and control regions (30–38% depending on lepton category), and from misidentification rate uncertainties (35–100% depending on lepton category).

**Fig. 2:**
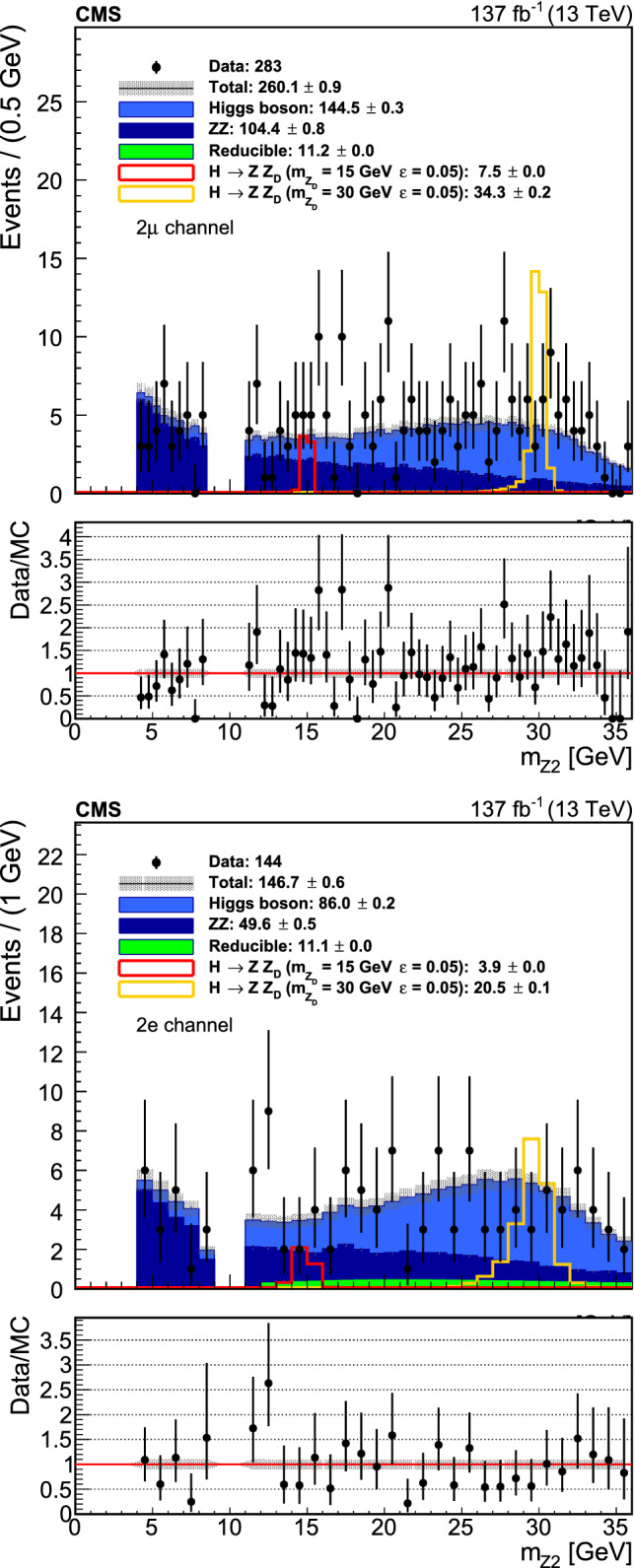
Event yields against $m_{{{\mathrm{Z}}} _2}$ with the ${{\mathrm{Z}}} {{\mathrm{X}}}$ selection for the muon and electron channels. Numbers in the legend show the total event yields with the ${{\mathrm{Z}}} {{\mathrm{X}}}$ selection corresponding to data, and the expected yields for each background and signal processes, along with the corresponding statistical uncertainty coming from the amount of simulated data

Theoretical uncertainties that affect both the signal and background estimation include uncertainties in the renormalization and factorization scales and the choice of the PDF set. The uncertainty from the renormalization and factorization scales is determined by varying these scales between 0.5 and 2 times their nominal value while keeping their ratio between 0.5 and 2. The uncertainty from the PDF set is determined by taking the root-mean-square of the variation when using different replicas of the default NNPDF set [64]. An additional uncertainty of 10% in the K factor used for the ${{\mathrm{g}}} {{\mathrm{g}}} \rightarrow 4\ell$ prediction is included [37]. To estimate the effect of the interference between the signal and background processes, three types of samples are generated using the MadGraph 5_amc@nlo 2.4.2 [39–41] generator: inclusive sample (${{{\mathrm{H}}} \rightarrow {{\mathrm{Z}}} {{\mathrm{Z}}} ^{*} \rightarrow 4\ell }$, ${{{\mathrm{H}}} \rightarrow {{\mathrm{Z}}} {{\mathrm{X}}}/ {{\mathrm{X}}} {{\mathrm{X}}} \rightarrow 4\ell }$), signal-only sample ${{{\mathrm{H}}} \rightarrow {{\mathrm{Z}}} {{\mathrm{X}}}/ {{\mathrm{X}}} {{\mathrm{X}}} \rightarrow 4\ell }$ and background-only sample ${{{\mathrm{H}}} \rightarrow {{\mathrm{Z}}} {{\mathrm{Z}}} ^{*} \rightarrow 4\ell }$. The inclusive sample contains background, signal, and interference contributions. The effect of the interference on the normalization of the signal is estimated by taking the difference of the inclusive sample cross section and the sum of the cross sections of the signal and background samples. This difference is at 1–2% after the final event selection. Theoretical values of branching fractions $\mathcal {B}({{\mathrm{Z}}} _{{\mathrm {D}}} \rightarrow {{\mathrm{e}}} {{\mathrm{e}}}\ \text {or}\ {{\mathrm{\upmu }}} {{\mathrm{\upmu }}})$ are calculated in Ref. [7]. The calculations are based on experimental measurements of the ratio of the hadronic cross section to the muon cross section in electron-positron collisions $R_{{{\mathrm{\upmu }}} {{\mathrm{\upmu }}}}/R_{\text {had}}$ up to $m_{{{\mathrm{Z}}} _{{\mathrm {D}}}} = 12\,\text {GeV}$ and a next-to-leading order theoretical calculation for $m_{{{\mathrm{Z}}} _{{\mathrm {D}}}} > 12\,\text {GeV}$. To account for uncertainties in these theoretical estimates, a conservative 20% (10%) uncertainty is assigned to them for $m_{{{\mathrm{Z}}} _{{\mathrm {D}}}} < 12\,\text {GeV}$ ($m_{{{\mathrm{Z}}} _{{\mathrm {D}}}} > 12\,\text {GeV}$) [7]. Differences in the kinematic properties between the HAHM and ALP model have been inspected. For the determination of model-independent exclusion limits, differences in acceptances are included as systematic uncertainties, ranging from 10% ($m_{{{\mathrm{X}}}} \sim 4\,\text {GeV}$) to 30% ($m_{{{\mathrm{X}}}} \sim 35\,\text {GeV}$ for ${{\mathrm{Z}}} {{\mathrm{X}}}$, $m_{{{\mathrm{X}}}} \sim 60\,\text {GeV}$ for ${{\mathrm{X}}} {{\mathrm{X}}}$), while they are used to correct signal yields for the determination of ALP exclusion limits.

In the combination of the three data taking periods, the theoretical uncertainties and experimental ones related to leptons are correlated across all data taking periods, while all others from experimental sources are taken as uncorrelated. The sensitivity of this analysis is dominated by data statistical uncertainty rather than systematic uncertainties.

## Results and interpretation

**Fig. 3:**
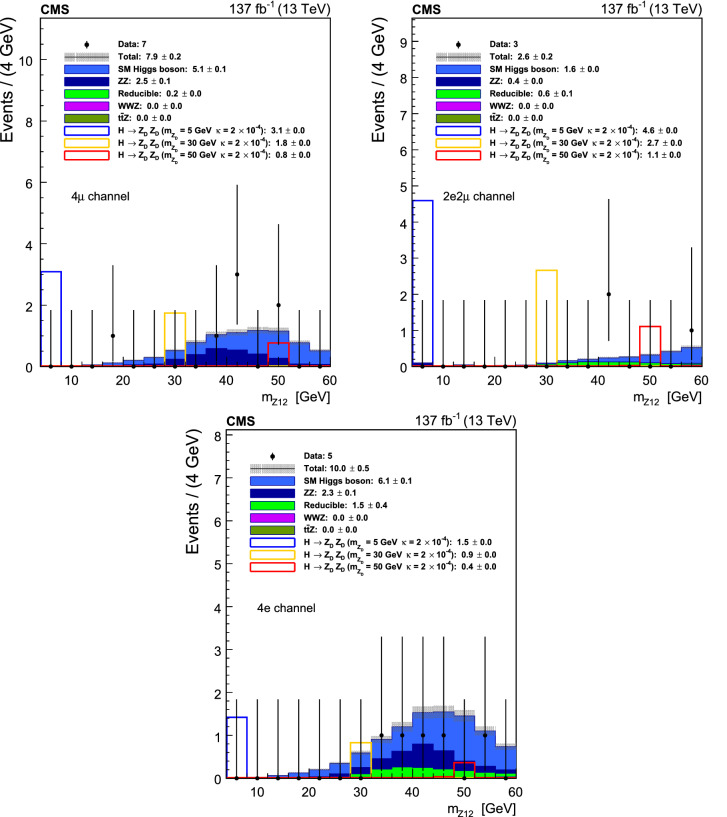
Event yields against $m_{Z12} = (m_{{{\mathrm{Z}}} _1} +m_{{{\mathrm{Z}}} _2})/2$ with the ${{\mathrm{X}}} {{\mathrm{X}}}$ selection for the 4$\mu$, 2e2$\mu$, and 4e final states. Numbers in the legend show the total event yields with the ${{\mathrm{X}}} {{\mathrm{X}}}$ selection corresponding to data, and the expected yields for each background and signal processes, along with the corresponding statistical uncertainty coming from the amount of simulated data

Dilepton mass distributions for the ${{\mathrm{Z}}} {{\mathrm{X}}}$ and ${{\mathrm{X}}} {{\mathrm{X}}}$ selections are shown in Figs. 2 and 3, respectively. The dilepton mass variable for the ${{\mathrm{X}}} {{\mathrm{X}}}$ selection shown in Fig. 3 is $m_{Z12} = (m_{{{\mathrm{Z}}} _1} +m_{{{\mathrm{Z}}} _2})/2$, which should peak at $m_{{{\mathrm{X}}}}$ in case of a signal ${{\mathrm{H}}} \rightarrow {{\mathrm{X}}} {{\mathrm{X}}}$. In all cases, the observed distributions agree well with standard model expectations within the assigned uncertainties.

These results are further interpreted as upper limits on model-independent branching fractions and model parameters for the dark photon and ALP models. For interpretations of the results of the ${{\mathrm{Z}}} {{\mathrm{X}}}$ selection, 351 mass hypotheses are considered. Each mass hypothesis $m_{i}$ is defined with an incremental step of 0.5%, as $m_{i} = 4.20 \times 1.005^{i}$, where $i = 0,1,2,\ldots ,424$, excluding the mass points around the $\varUpsilon$ ${{\mathrm{b}}} {{{\overline{\mathrm{{{\mathrm{b}}}}}}}}$ bound states between $8.0< m_{\varUpsilon } < 11.5\,\text {GeV}$ ($i = 130,131,\ldots ,201$). The incremental step is chosen so as not to miss any potential signal contribution due to detector resolution in $m_{{{\mathrm{Z}}} _2}$. For each mass hypothesis, the counting experiments are performed on the $m_{{{\mathrm{Z}}} _2}$ distribution, with the bin centered at each mass hypothesis. Because of the finite mass resolution of $m_{{{\mathrm{Z}}} _2}$, the choice of the bin width needs to be defined such that most of the signal contribution is included in the bin. The bin width is defined as $0.04\,(0.10) \times m_{i}$ for the $4{{\mathrm{\upmu }}}$ and $2{{\mathrm{e}}} 2{{\mathrm{\upmu }}}$ ($4{{\mathrm{e}}}$ and $2{{\mathrm{\upmu }}} 2{{\mathrm{e}}}$) categories. This width is chosen as two times the $m_{{{\mathrm{Z}}} _2}$ resolution and includes $\approx$95% of signal events. The normalization of the Higgs background is allowed to float freely in the likelihood fit. For each mass hypothesis, events outside the mass window are included as a sideband to constrain the normalization parameter. No significant deviation with respect to the SM prediction is observed.

For interpretations of the results of the ${{\mathrm{X}}} {{\mathrm{X}}}$ selection, 462 mass hypotheses are considered instead. In contrast to the ${{\mathrm{Z}}} {{\mathrm{X}}}$ interpretations, the counting experiments are performed by constructing a rectangular region, centered at each mass hypothesis, in the ($m_{{{\mathrm{Z}}} _1}$,$m_{{{\mathrm{Z}}} _2}$) plane. The rectangular regions are effectively triangular as $m_{{{\mathrm{Z}}} _1}$ is defined as the larger invariant mass. The bin widths are defined in a similar manner as $0.04 m_{i}$ ($0.10 m_{i}$) for $m_{{{\mathrm{Z}}} _1}$ or $m_{{{\mathrm{Z}}} _2}$ formed by muon (electron) pairs.

The likelihood model for each mass hypothesis is formulated as

3 $$\begin{aligned} \mathcal {L}_{m}= & {} \mathcal {L}_{m,\mathrm {SR}} \mathcal {L}_{m,\text {SB}} \end{aligned}$$

4 $$\begin{aligned} \mathcal {L}_{m,\mathrm {SR}}= & {} \prod _{\ell } {{\,\mathrm{Pois}\,}}( n_{m,\ell } | \mu _{\text {Higgs}} n_{\text {Higgs},m,\ell } \nonumber \\&+ \sum _{b} n_{b,m,\ell } \rho _{b,m,\ell } + \mu n_{s,m,\ell } \rho _{s,m,\ell }), \end{aligned}$$

5 $$\begin{aligned} \mathcal {L}_{m,\text {SB}}= & {} \prod _{\ell } {{\,\mathrm{Pois}\,}}( n_{\ell } | \mu _{\text {Higgs}} n_{\text {Higgs},\ell } + \sum _{b} n_{b,\ell } \rho _{b,\ell } ) \end{aligned}$$

where the function ${{\,\mathrm{Pois}\,}}(n|x)$ is the Poisson probability to observe *n* events, when the expectation is *x*. The symbol *m* represents a particular mass hypothesis. The likelihood term $\mathcal {L}_{m,{\mathrm {SR}}}$ ($\mathcal {L}_{m,\text {SB}}$) corresponds to the event yields within (outside) the mass window. The symbol $\mu$ is the signal strength parameter, $\mu _{\text {Higgs}}$ represents the free floating normalizing parameter on the SM Higgs boson process, $\ell$ represents each lepton category, *b* represents each background process, *s* represents a particular signal process and $n_{i,m,\ell }$ represents the yield in a mass window associated with the mass hypothesis *m*, from a source *i* and the lepton category $\ell$. In Eq. (5), the symbols $n_{\text {Higgs},\ell }$ and $n_{b,\ell }$ represent the yields of the SM Higgs boson and other backgrounds *b* outside the mass window for the lepton category $\ell$. Systematic uncertainties are included and profiled as nuisance parameters $\rho$ [65].

For each interpretation, $95\%$ exclusion limits are obtained with an asymptotic formulation of the modified frequentist $\text {CL}_\text {s}$ criterion as described in Refs. [65–68] with the ZX selection and full $\text {CL}_\text {s}$ approach for the XX selection.

### Model-independent limits

**Fig. 4:**
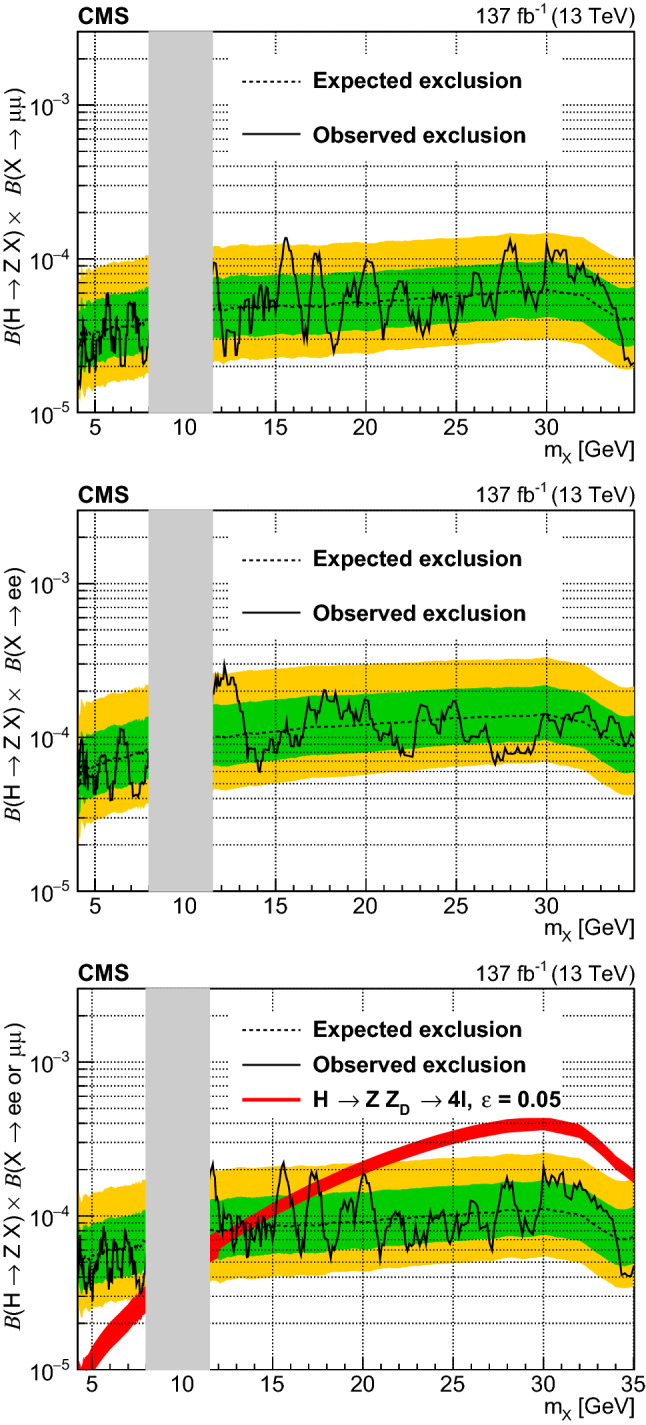
Expected and observed 95% CL limits on $\mathcal {B}({{\mathrm{H}}} \rightarrow {{\mathrm{Z}}} {{\mathrm{X}}}) \mathcal {B}({{\mathrm{X}}} \rightarrow {{\mathrm{\upmu }}} {{\mathrm{\upmu }}})$ assuming ${\mathrm{X}}$ decays to dimuons only, $\mathcal {B}({{\mathrm{H}}} \rightarrow {{\mathrm{Z}}} {{\mathrm{X}}}) \mathcal {B}({{\mathrm{X}}} \rightarrow {{\mathrm{e}}} {{\mathrm{e}}})$ assuming ${\mathrm{X}}$ decays to dielectrons only, and $\mathcal {B}({{\mathrm{H}}} \rightarrow {{\mathrm{Z}}} {{\mathrm{X}}}) \mathcal {B}({{\mathrm{X}}} \rightarrow {{\mathrm{e}}} {{\mathrm{e}}}\ \text {or}\ {{\mathrm{\upmu }}} {{\mathrm{\upmu }}})$ assuming a flavor symmetric decay of ${\mathrm{X}}$ to dimuons and dielectrons. The dashed black curve is the expected upper limit, with one and two standard-deviation bands shown in green and yellow, respectively. The solid black curve is the observed upper limit. The red curve represents the theoretical cross section for the signal process ${{\mathrm{H}}} \rightarrow {{\mathrm{Z}}} {{\mathrm{X}}} \rightarrow 4\ell$. The discontinuity at 12$\,\text {GeV}$ in the uncertainty is due to the switch from experimental to theoretical uncertainty estimates of $\mathcal {B}({{\mathrm{Z}}} _{{\mathrm {D}}} \rightarrow {{\mathrm{e}}} {{\mathrm{e}}}\ \text {or}\ {{\mathrm{\upmu }}} {{\mathrm{\upmu }}})$, as described in Ref. [7]. The symbol $\varepsilon$ is the kinetic-mixing parameter. The grey band corresponds to the excluded region around the ${{\mathrm{b}}} {{{\overline{\mathrm{{{\mathrm{b}}}}}}}}$ bound states of $\varUpsilon$

**Fig. 5:**
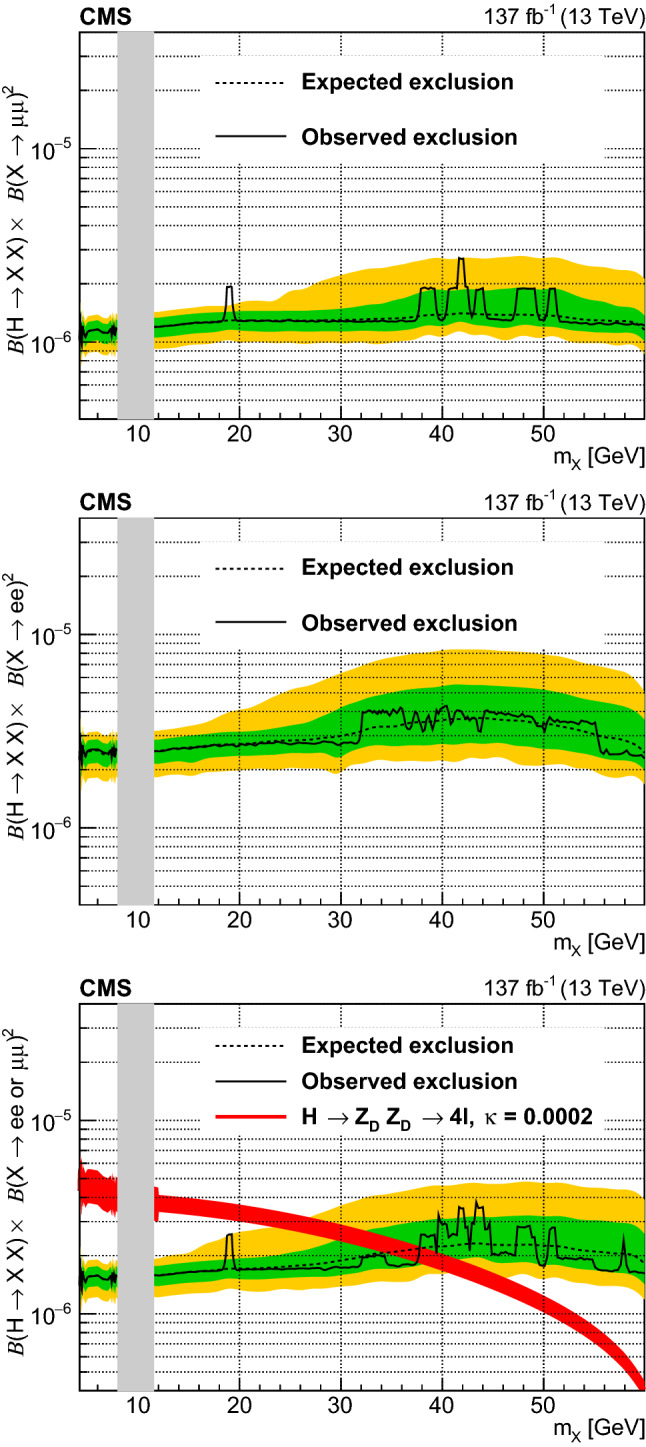
Expected and observed 95% CL limits on $\mathcal {B}({{\mathrm{H}}} \rightarrow {{\mathrm{X}}} {{\mathrm{X}}}) \mathcal {B}({{\mathrm{X}}} \rightarrow {{\mathrm{\upmu }}} {{\mathrm{\upmu }}})^2$ assuming ${\mathrm{X}}$ decays to dimuons only, $\mathcal {B}({{\mathrm{H}}} \rightarrow {{\mathrm{X}}} {{\mathrm{X}}}) \mathcal {B}({{\mathrm{X}}} \rightarrow {{\mathrm{e}}} {{\mathrm{e}}})^2$ assuming ${\mathrm{X}}$ decays to dielectrons only, and $\mathcal {B}({{\mathrm{H}}} \rightarrow {{\mathrm{X}}} {{\mathrm{X}}}) \mathcal {B}({{\mathrm{X}}} \rightarrow {{\mathrm{e}}} {{\mathrm{e}}}\ \text {or}\ \mu \mu )^2$ assuming a flavor symmetric decay of ${\mathrm{X}}$ to dimuons and dielectrons. The dashed black curve is the expected upper limit, with one and two standard-deviation bands shown in green and yellow, respectively. The solid black curve is the observed upper limit. The red curve represents the theoretical cross section for the signal process ${{\mathrm{H}}} \rightarrow {{\mathrm{X}}} {{\mathrm{X}}} \rightarrow 4\ell$. The discontinuity at 12$\,\text {GeV}$ in uncertainty is due to the switch from experimental to theoretical uncertainty estimates of $\mathcal {B}({{\mathrm{Z}}} _{{\mathrm {D}}} \rightarrow {{\mathrm{e}}} {{\mathrm{e}}}\ \text {or}\ {{\mathrm{\upmu }}} {{\mathrm{\upmu }}})$, as described in Ref. [7]. The symbol $\kappa$ is the Higgs-mixing parameter. The grey band corresponds to the excluded region around the ${{\mathrm{b}}} {{{\overline{\mathrm{{{\mathrm{b}}}}}}}}$ bound states of $\varUpsilon$

Upper limits at 95% confidence level (CL) are derived on model-independent branching fractions with the ${{\mathrm{Z}}} {{\mathrm{X}}}$ and ${{\mathrm{X}}} {{\mathrm{X}}}$ selections assuming three decay channels: a flavor symmetric decay of ${\mathrm{X}}$ to a muon or an electron pair, exclusive ${\mathrm{X}}$ decays to a muon pair, and exclusive ${\mathrm{X}}$ decays to an electron pair. Acceptance effects arising from different signal models are included as systematic uncertainties in the signal yields after event selection. Little model dependence is expected as the event selection is defined without using angular information between the leptons. Figures 4 and 5 show the exclusion limits on the model-independent branching fractions with the ${{\mathrm{Z}}} {{\mathrm{X}}}$ and ${{\mathrm{X}}} {{\mathrm{X}}}$ selections, respectively. The weaker observed limit in the XX selection at ${m_{{{\mathrm{X}}}} \approx 18\,\text {GeV}}$ is due to one observed data event and does not represent a significant statistical deviation from the background hypothesis. Kinematic differences between the dark photon and ALP models are included as systematic uncertainties, as detailed in Sect. 6.

### Limits on dark photon model parameters

**Fig. 6:**
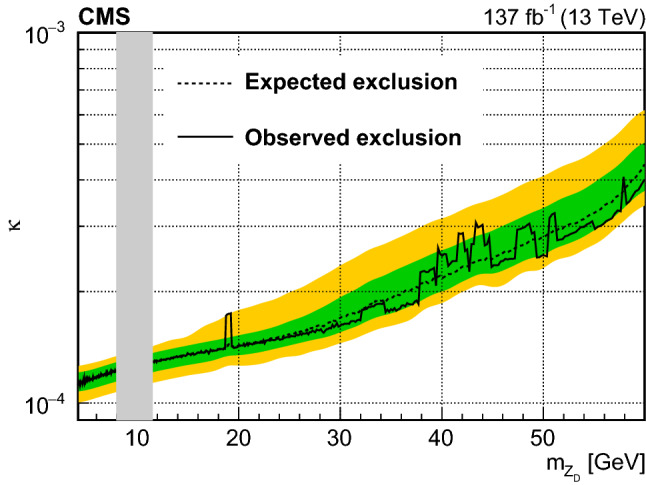
95% CL limits on the Higgs-mixing parameter $\kappa$, based on the ${{\mathrm{X}}} {{\mathrm{X}}}$ selection, as function of $m_{{{\mathrm{Z}}} _{{\mathrm {D}}}}$. The dashed black curve is the expected upper limit, with one and two standard-deviation bands shown in green and yellow, respectively. The solid black curve is the observed upper limit. The grey band corresponds to the excluded region around the ${{\mathrm{b}}} {{{\overline{\mathrm{{{\mathrm{b}}}}}}}}$ bound states of $\varUpsilon$

Upper limits at 95% CL are obtained on the Higgs-mixing parameter $\kappa$ and ${\mathcal {B}({{\mathrm{H}}} \rightarrow {{\mathrm{Z}}} _{{\mathrm {D}}} {{\mathrm{Z}}} _{{\mathrm {D}}})}$ with the ${{\mathrm{X}}} {{\mathrm{X}}}$ selection, as shown in Fig. 6, assuming $\kappa \gg \varepsilon$. The LHC provides unique sensitivity to the parameter $\kappa$ due to the presence of the Higgs boson. In addition, this analysis provides some sensitivity to $\varepsilon$, but the upper limits are almost an order of magnitude weaker than those from the Drell–Yan search and from the LHCb Collaboration [14], and hence are not reported in this paper.

### Limits on the ALP model

**Fig. 7:**
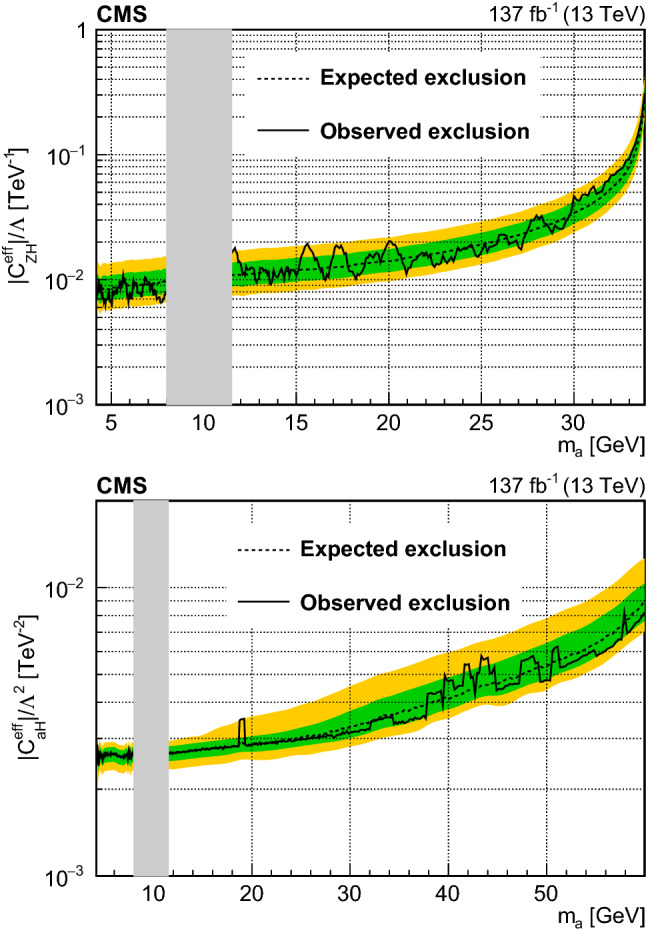
95% CL limit on $C_{{{\mathrm{Z}}} {{\mathrm{H}}}}/\varLambda$ and $C_{{{\mathrm{a}}} {{\mathrm{H}}}}/\varLambda ^2$ as function of $m_{{{\mathrm{a}}}}$. Black curves are the expected upper limits, with one and two standard-deviation bands shown in green and yellow, respectively. The solid black curves represent the observed upper limits. The grey band corresponds to the excluded region around the ${{\mathrm{b}}} {{{\overline{\mathrm{{{\mathrm{b}}}}}}}}$ bound states of $\varUpsilon$

Upper limits at 95% CL are calculated on the Wilson coefficients $C_{{{\mathrm{Z}}} {{\mathrm{H}}}}/\varLambda$ and $C_{{{\mathrm{a}}} {{\mathrm{H}}}}/\varLambda ^2$, as shown in Fig. 7, where $C_{{{\mathrm{Z}}} {{\mathrm{H}}}}$ is the effective coupling parameter of the Higgs boson, ${\mathrm{Z}}$ boson, and the ALP, $C_{{{\mathrm{a}}} {{\mathrm{H}}}}$ is the effective coupling parameter of the Higgs boson and the ALP, and $\varLambda$ is the new physics scale. In both interpretations, the ALP is assumed to decay promptly with $\mathcal {B}({{\mathrm{a}}} \rightarrow {{\mathrm{e}}} {{\mathrm{e}}}\ \text {or}\ {{\mathrm{\upmu }}} {{\mathrm{\upmu }}}) = 1$, with equal fractions to muons and electrons. The last six mass hypotheses are omitted in the calculation of upper limits on $C_{{{\mathrm{Z}}} {{\mathrm{H}}}}/\varLambda$ to match the $m_{{{\mathrm{a}}}}$ range adopted in Ref. [20]. Kinematic differences between the dark photon and ALP models are included as corrections on signal region yields, as detailed in Sect. 6.

## Summary

A search for dilepton resonances in Higgs boson decays to four-lepton final states has been presented. The search considers the two intermediate decay topologies ${{\mathrm{H}}} \rightarrow {{\mathrm{Z}}} {{\mathrm{X}}}$ and ${{\mathrm{H}}} \rightarrow {{\mathrm{X}}} {{\mathrm{X}}}$. No significant deviations from the standard model expectations are observed. The search imposes experimental constraints on products of model-independent branching fractions of $\mathcal {B}({{\mathrm{H}}} \rightarrow {{\mathrm{Z}}} {{\mathrm{X}}})$, $\mathcal {B}({{\mathrm{H}}} \rightarrow {{\mathrm{X}}} {{\mathrm{X}}})$ and $\mathcal {B}({{\mathrm{X}}} \rightarrow {{\mathrm{e}}} {{\mathrm{e}}}\ \text {or}\ {{\mathrm{\upmu }}} {{\mathrm{\upmu }}})$, assuming flavor-symmetric decays of ${\mathrm{X}}$ to dimuons and dielectrons, exclusive decays of ${\mathrm{X}}$ to dimuons, and exclusive decays of ${\mathrm{X}}$ to dielectrons, for $m_{{{\mathrm{X}}}} > 4\,\text {GeV}$. In addition, two well-motivated theoretical frameworks beyond the standard model are considered. Due to the presence of the Higgs boson production in LHC proton–proton collisions, the search provides unique constraints on the Higgs-mixing parameter $\kappa < 4 \times 10^{-4}$ at 95% confidence level ($\text {CL}$) in a dark photon model with the ${{\mathrm{X}}} {{\mathrm{X}}}$ selection, in Higgs-mixing-dominated scenarios, while searches for ${{\mathrm{Z}}} _{{\mathrm {D}}}$ in Drell–Yan processes [14, 69] provide better exclusion limits on $\varepsilon$ in kinetic-mixing-dominated scenarios. For the axion-like particle model, upper limits at 95% CL are placed on two relevant Wilson coefficients $C_{{{\mathrm{Z}}} {{\mathrm{H}}}}/\varLambda$ and $C_{{{\mathrm{a}}} {{\mathrm{H}}}}/\varLambda ^2$. This is the first direct limit on decays of the observed Higgs boson to axion-like particles decaying to leptons.

## Data Availability

This manuscript has no associated data or the data will not be deposited. [Authors’ comment: For CMS Release and preservation of data used by the CMS Collaboration as the basis for publications is guided by the CMS policy as stated in “CMS data preservation, re-use and open access policy” (https://cms-docdb.cern.ch/cgi-bin/PublicDocDB/RetrieveFile?docid=6032&filename=CMSDataPolicyV1.2.pdf&version=2).]
